# Boosting Lattice Oxygen Oxidation of Perovskite to Efficiently Catalyze Oxygen Evolution Reaction by FeOOH Decoration

**DOI:** 10.34133/2020/6961578

**Published:** 2020-07-10

**Authors:** Jia-Wei Zhao, Cheng-Fei Li, Zi-Xiao Shi, Jie-Lun Guan, Gao-Ren Li

**Affiliations:** MOE Laboratory of Bioinorganic and Synthetic Chemistry, The Key Lab of Low-Carbon Chemistry & Energy Conservation of Guangdong Province, School of Chemistry, Sun Yat-sen University, Guangzhou 510275, China

## Abstract

In the process of oxygen evolution reaction (OER) on perovskite, it is of great significance to accelerate the hindered lattice oxygen oxidation process to promote the slow kinetics of water oxidation. In this paper, a facile surface modification strategy of nanometer-scale iron oxyhydroxide (FeOOH) clusters depositing on the surface of LaNiO_3_ (LNO) perovskite is reported, and it can obviously promote hydroxyl adsorption and weaken Ni-O bond of LNO. The above relevant evidences are well demonstrated by the experimental results and DFT calculations. The excellent hydroxyl adsorption ability of FeOOH-LaNiO_3_ (Fe-LNO) can obviously optimize OH^−^ filling barriers to promote lattice oxygen-participated OER (LOER), and the weakened Ni-O bond of LNO perovskite can obviously reduce the reaction barrier of the lattice oxygen participation mechanism (LOM). Based on the above synergistic catalysis effect, the Fe-LNO catalyst exhibits a maximum factor of 5 catalytic activity increases for OER relative to the pristine perovskite and demonstrates the fast reaction kinetics (low Tafel slope of 42 mV dec^−1^) and superior intrinsic activity (TOFs of ~40 O_2_ S^−1^ at 1.60 V vs. RHE).

## 1. Introduction

Oxygen evolution reaction (OER) as a half reaction of overall water splitting is important for many energy storage applications such as solar cells, metal-air batteries, and fuel cell [1–3]. However, its sluggish kinetics with multistep proton-coupled electron transfer and high overpotential frustrate its commercial applications. Though the state-of-the-art precious metal oxides such as IrO_2_ or RuO_2_ demonstrate the most active OER catalysts, they suffer significantly from high cost and instability in the practical applications [4]. In recent studies, emerging as a family of outstanding alternatives, crystalline or amorphous transitional metal oxides and metal oxyhydroxides have been widely exploited because of their high catalytic activity, low cost, and environmentally benign character [5]. Among them, low-cost perovskite with ABO_3_ formula (A is a rare-earth or alkaline-earth element and B is a transition metal) has drawn particular attention due to its structural adjustability and high intrinsic performance [6–8]. For example, Suntivich et al. proposed a structural regulation method to optimize the OER activity of perovskite by tailoring the e_g_ filling electrons of B site transition metal [9]. Grimaud et al. reported that the OER catalytic performance of double perovskites is greatly related to the lattice oxygen 2p band center [10]. Despite the fact that much progress has been made on establishing related theory to explain the OER activity of perovskites, there are still some challenging facts including (1) explanation of the nonconcerted proton-electron transfer (NCPET) process of some perovskites during OER, which exhibits pH-dependent catalytic activity on the RHE scale; (2) the surface structural change with A site leaching during OER; and (3) the OER performance related to the O vacancies and O 2p band center in perovskite. Therefore, it is highly urgent to propose an effective theory to comprehensively understand the OER mechanism of perovskite.

With further studies of perovskite in recent years, it has been found that the mechanism of the NCPET process and surface structural change of perovskite are attributed to the lattice oxygen-participated OER (LOER), which was first observed by in situ ^18^O isotope labeling with mass spectrometry [11]. Additionally, numerous studies have indicated that the unusual reaction pathway of the lattice oxygen participation mechanism (LOM) has a faster OER kinetics and lower reaction energy barriers compared with adsorbate evolution mechanism (AEM) [12–14], because of bypassing the theoretical overpotential limitation of 0.37 V [15]. Therefore, the study of LOER is of great significance for perovskites. To the best of our knowledge, one of the most direct ways to active LOER of perovskite is replacing the alkaline-earth metal A site to regulate the strength of metal-oxygen bond [11]. For example, Mefford et al. found that LaCoO_3-*δ*_ underwent LOER via Sr doping (Sr > 50%) [16], which showed excellent OER performance. However, this method is not applicable to other perovskites such as LaNiO_3-*δ*_, which lead to structural transformation and performance degradation after Sr doping [17]. Therefore, to overcome this shortcoming, a new method should be proposed to boost the LOER of perovskite without changing the original structure of catalysts.

With this intention, we focus on the key step of LOM based on our previous studies [18–35], a OH^−^ filling pathway, as shown in the following formulas {[O = O‐MO_4_] → [V‐MO_4_] + *e*^−^} and {[V‐MO_4_] + OH^−^ → [HO_ads_‐MO_4_] + *e*^−^}, where M, [MO_4_], and V denote the transition metal B site of perovskite, the surface structure with oxygen vacancies, and O vacancy, respectively. Some studies have demonstrated that OH^−^ filling is thermodynamically favorable in some perovskite and plays a key role in LOER [36]. However, the OH^−^ filling is much slower than leaching of lattice oxygen [37], which restricts the performance and even causes intensive surface structural change during OER [38]. Therefore, accelerating the lattice oxygen participation process of perovskite and promoting hydroxyl adsorption will be a direct and efficient method to enhance the catalytic performance of perovskite, but this method is almost a blank field to date. Based on this assumption, we present a facile surface modification strategy to directly optimize the hydroxyl adsorption step on oxygen vacancy of B site of perovskite to accelerate the lattice oxygen participation process by depositing FeOOH nanoclusters on the surface of LaNiO_3_ (LNO) perovskite (a representative semimetallic perovskite). As we all know, metal oxyhydroxides own high hydroxyl adsorption capacity [39–41]. Furthermore, the electronic effect between metal oxyhydroxide and metal oxide will change the chemical environments of the interfaces [42]. The excellent hydroxyl adsorption ability of FeOOH-LaNiO_3_ (Fe-LNO) can obviously accelerate LOER, while the strong electronic interaction between FeOOH and LNO perovskite can lead to positive shifting of the O 2p band center and negative shifting of the Ni 3d center of LNO, which will obviously reduce the reaction barrier of LOM. The above advantages make Fe-LNO own high catalytic performance for OER, which is comparable to that of state-of-the-art Ni-Fe (oxy)hydroxide amorphous catalysts. This work provides a novel strategy to optimize and study the LOER of perovskites.

## 2. Results and Discussion

### 2.1. Fe-LNO Catalyst Synthesis and Characterizations

**Figure 1:**
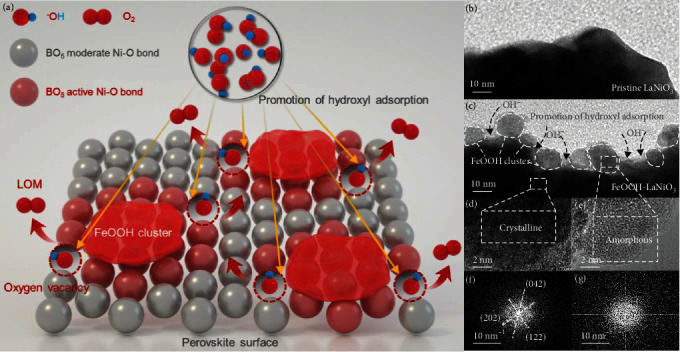
Catalyst synthesis and characterizations. (a) The illustration of synergetic catalysis mechanism of Fe-LNO. (b, c) TEM image of pristine LNO and Fe-LNO. (d, e) HRTEM images of LNO and Fe-LNO. (f, g) The corresponding FFT images of LNO and Fe-LNO.

The heterostructured Fe-LNO was synthesized by a simple deposition way. Firstly, polycrystalline LaNiO_3_ (LNO) powders were synthesized from a mixture of metal nitrate (La and Ni) and citric acid through the Pechini method. Secondly, the obtained LNO powders were dispersed in Fe^2+^ solution of low concentration, and the pH of the solution was kept around 4 to prevent LNO from etching (Figure S1) [43]. After deposition of FeOOH for 5 min, the Fe-LNO sample was obtained by centrifuging the above solution three times. Fundamental characterizations of LNO and Fe-LNO by scanning electron microscopy (SEM) and powder X-ray diffraction (PXRD) were performed. The samples LNO and Fe-LNO both exhibit the nanoparticle assemble morphology with a similar size distribution in the range of 100 to 150 nm as shown in Figures S2 and S3. In addition, the Rietveld refined XRD patterns in Figures S8 and S9 indicate the rhombohedral structure (space group: R3̅c) of LNO with a lattice constant of *a* = *b* = 5.457 Å, *c* = 13.180 Å, and there is no extra peak for the Fe-LNO sample, which shows that FeOOH is amorphous. Furthermore, the structure of FeOOH nanoclusters was confirmed by Raman spectra and high-resolution X-ray photoelectron spectroscopy (XPS) spectra (Figures S10b and S14b). The Raman peaks at around 560 cm^−1^ are ascribed to the typical *α*-FeOOH structure [44], and the XPS peak of Fe 2p also confirmed the existence of FeOOH [45]. To characterize the contact state of Fe-LNO two phases, the transmission electron microscopy (TEM) images of LNO and Fe-LNO are shown in Figures 1(b) and 1(c), which show that the pristine LNO has a smooth surface, while FeOOH nanoparticles with ~10 nm are discontinuously distributed on the LNO surface. A high density of heterointerfaces of FeOOH/LNO is obtained. To further confirm the compositions of two phases, high-resolution TEM (HRTEM) images are shown in Figure 1(d), which shows that the structure of inner LNO is crystalline, while FeOOH nanoparticles are amorphous (Figure 1(e)). The heterointerfaces of FeOOH/LNO can be further confirmed by fast Fourier transform (FFT) as shown in Figures 1(f) and 1(g), which also indicates polycrystalline LNO and amorphous FeOOH nanoparticles, respectively.

Given the unique structure of Fe-LNO and the synergistic effect of heterointerface, we propose the synergetic catalysis mechanism to regulate LOER as schematically illustrated in Figure 1(a). The LNO is the main catalysis site, and the FeOOH nanoclusters served as a cocatalyst for promoting hydroxyl adsorption. Beyond that, under the synergistic effect of heterointerface, the FeOOH nanoclusters can weaken the Ni-O bond in LNO to facilitate the formation of O_NB_, which is preferred to be oxidized during water oxidation and will reduce the energy barrier of LOER [15].

### 2.2. Theoretical Study of LOM Promoted by FeOOH

**Figure 2:**
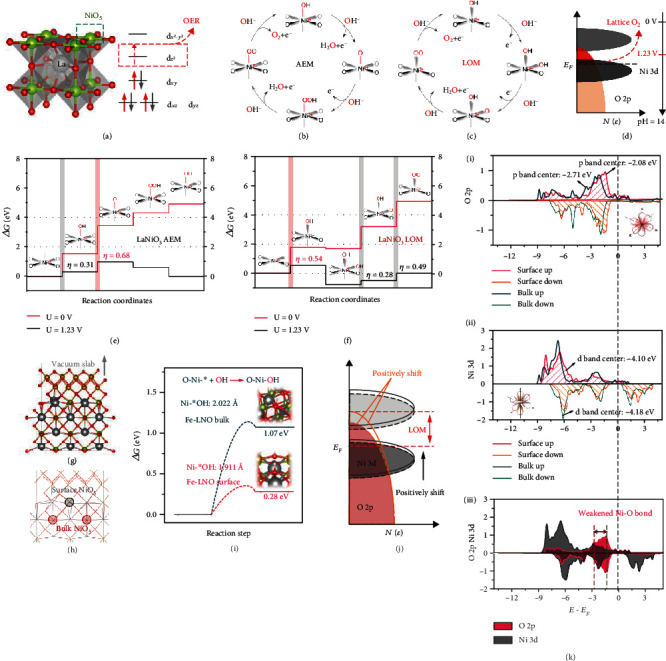
Theoretical studies of LOM and AEM of Fe-LNO. (a) The ABO_3_ structures with chemical reaction surface [BO_5_]. (b, c) The proposed OER mechanisms of LNO, including AEM and LOM. (d) Schematic rigid band diagrams of LNO. The position of the O_2_/H_2_O redox couple at pH 14 is 1.23 V versus RHE. (e, f) Free energies of OER steps via AEM and LOM mechanisms on LNO. (g, h) The constructed model of Fe-LNO heterostructure. (i) The hydroxl adsorption energy of LNO and Fe-LNO. (j) LOM mechanism optimized by FeOOH. (k) pDOS of O(2p) and Ni(3d) orbitals in the surface NiO_5_ and bulk NiO_5_.

As we all know, the semimetallic Ni-based crystalline oxide LaNiO_3_ (LNO) exhibiting high OER activity mainly arises from the structure and molecular orbital of LNO [13]. Namely, the Ni cations are in the low spin state with fully occupied t_2g_ states (Figures S15–S17), and the e_g_ orbit of Ni^3+^ participates in *σ*-bonding with the surface-anion adsorbate, accompanied with the single electron and high energy of e_g_ orbit promoting OER catalytic activity [9]. However, according to recent studies, the high OER catalytic activity of LNO is caused by the lattice oxygen redox reaction [12, 36]. When the O 2p state at the LNO Fermi level lies above the oxidation energy of H_2_O, the LOM step in the perovskites becomes thermodynamically favorable (Figure 2(d)) [46]. Therefore, according to the above-mentioned methods, the studies are mainly focused on the LOM and AEM [11]. The main difference between LOM and AEM lies in the coordination mode of hydroxyl groups: AEM is the top of the B site (Figure 2(b)), while LOM is the top of the B site near the oxygen vacancy (Figure 2(c)). In addition, during the AEM process, transition metal oxides are often limited by the second step of OER for the large O∗ formation energy (G_O__∗_) [47, 48] while the barrier of LOM is the hydroxyl adsorption step (G_OH__∗_) [12]. Therefore, we further simulate different mechanisms of LNO by the density functional theory (DFT) calculations as shown in Figures 2(e) and 2(f), and the related reaction structures are listed in Figures S21 and S22. The OER pathway free energy of 4-coordinated Ni is always lower than that of 5-coordinated Ni except the hydroxyl adsorption step, which can be attributed to the decrease of dz^2^ energy [49] and the easier formation of Ni-O bond during OER, resulting to the fact that the theoretical overpotential of LOM is much lower than that of AEM. However, the actual operation activity of LNO is much lower than the predicted results [50, 51], and this can be mainly ascribed to two reasons: (1) the barrier of hydroxyl adsorption and (2) the stable Ni-O bond that obstructs the formation of the O_NB_ state. In order to address the above questions, we first study the electronic effect between FeOOH and LNO by the first principle DFT calculations. The typical model of Fe-LNO is shown in Figures 2(g) and 2(h) and Figure S26. As the structure of FeOOH nanoclusters is too large for a plane wave-based periodicity DFT simulations [52], the interaction between two catalysts is constructed by supercell matching [53]. The lattice mismatching error is less than 8%, and the whole structure is completely relaxed to ensure no additional lattice stress. In order to compare the electronic effects between two phases, NiO_5_ in the adjacent FeOOH layer is marked as the surface layer, and the NiO_5_ in the interior is marked as bulk. Based on the above model, the synergistic effects between FeOOH and LNO are studied in Figure 2(i). It is evident that the *G*(∗OH) value (0.28 eV) of Fe-LNO catalyst is significantly lower than that of LNO (1.07 eV), suggesting that the FeOOH can facilitate hydroxyl adsorption on the surface of LNO, which is consistent with the differential charge transfer analysis as shown in Figure S28. Ni 3d electron pairs induced by FeOOH make the hydroxyl coordination more stable and thus shorten Ni-OH bond from 2.022 Å to 1.911 Å. Furthermore, the calculated projected density of states (PDOS) of surface NiO_5_ and bulk NiO_5_ has been conducted as shown in Figures 2(j) and 2(k). Both of the centers of Ni 3d band and O 2p band shift positively, making the lattice oxygen closer to the Fermi level. The lattice O in NiO_5_ will become more favorable to be oxidized during OER, and the surface Ni will become more easy to coordinate with ∗OH. These results demonstrate that the FeOOH can optimize OH^−^ filling barriers and weaken Ni-O bond of LNO to decrease the energy barrier of LOM and promote reaction kinetics.

### 2.3. The Evidences of Hydroxyl Adsorption Promoted by FeOOH

**Figure 3:**
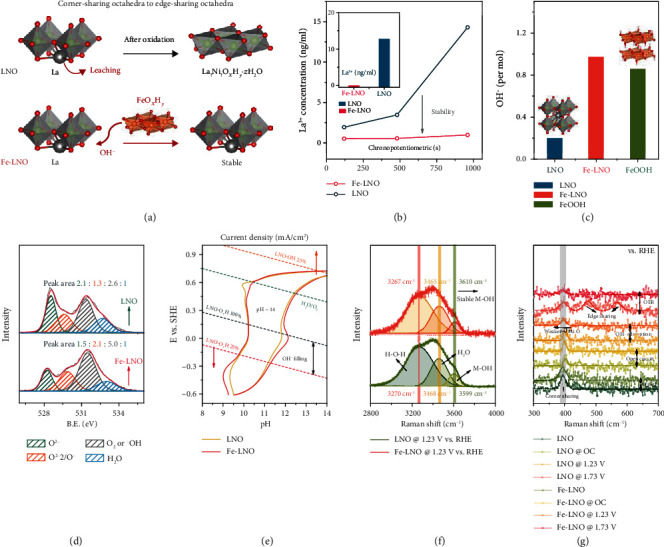
Evidence of hydroxyl adsorption promotion by FeOOH. (a) Illustration of the mechanism of hydroxyl adsorption with structure change. (b) Time-dependent ICP-MS and ICP-AES of LNO and Fe-LNO; test with chronopotentiometry holds at 10 mA/cm^2^. (c) Hydroxyl adsorption experiment by pH study. (d) O 2p XPS spectra of the Fe-LNO and LNO. (e) Cyclic voltammograms and hydroxyl adsorption voltage ranges by DFT calculations; the solid line represents the cyclic voltammograms of samples, and the dashed line is the prediction voltage ranges. (f) In situ Raman spectra of Fe-LNO and LNO at 1.23 V vs. RHE. (g) In situ Raman spectra of Fe-LNO and LNO in different voltages.

The leaching of A site, resulting in mass loss of La in LNO, is a universal phenomenon during the LOER. This phenomenon is due to the lower barrier of hydroxyl adsorption than that of lattice oxygen leaching [4, 37, 54], as shown in Figure 3(a). Therefore, this phenomenon will be significantly weakened with the acceleration of hydroxyl adsorption by FeOOH. In order to prove the hydroxyl adsorption mechanism, the time-dependent test was carried out with an inductively coupled plasma-mass spectrometer (ICP-MS) and inductively coupled plasma-atomic emission spectrometry (ICP-AES) as shown in Figure 3(b). We detected the La concentration in the electrolyte at different chronopotentiometric times. The amount of La leached from the LNO sample is 14.3 ng ml^−1^ (ICP-MS) and 12.5 ng ml^−1^ (ICP-AES), while the amount of La leached from the Fe-LNO sample is 0.98 ng ml^−1^ (ICP-MS) and 0 ng ml^−1^ (ICP-AES), indicating that the FeOOH can effectively alleviate the leaching of La. This result well proves the promotion of hydroxyl adsorption. To further demonstrate that FeOOH can promote the hydroxyl adsorption of LNO, we also used a simple pH test to measure the change of pH in the supernatant by dispersing the material evenly in the same solution, and then, we calculated the hydroxyl adsorption of catalyst (Figure 3(c)). The Fe-LNO exhibits the highest hydroxyl adsorption capacity nearly 1 : 1, and the hydroxyl adsorption capacity of Fe-LNO is even better than that of pure FeOOH, which is much larger than that of LNO (0.2 : 1), indicating that the modification of FeOOH can greatly optimize the adsorption of hydroxyl. To further determine whether the adsorbents are chemisorption of ∗OH or physisorption of hydroxyl, XPS was used to further prove the adsorption state of hydroxyl [55], as shown in Figure 3(d). By comparing the area ratio of the hydroxyl peak and water peak of Fe-LNO and LNO, we found that the adsorption of hydroxyl by Fe-LNO was significantly improved in the range of 2.6 : 1 to 5.0 : 1, implying the generation of a high density of extra oxygen vacancies filled by hydroxyl. We also compared the hydroxyl peak ratio of pure FeOOH in Figure S14a, which is in the range of 3.5 : 1, less than that of Fe-LNO. In addition, the peak at around 529.5 eV can be attributed to the O_NB_ states [15], which indicates that the FeOOH can activate more O_NB_ states, and this is consistent with the DFT calculation results.

Furthermore, in order to display that hydroxyl adsorption mainly occurred at the electric field, the cyclic voltammetry and in situ Raman spectroscopy were measured. Cyclic voltammetry is a powerful tool for analyzing the chemisorption and oxidation of catalysts, which are widely utilized in Pt, Au, RuO_2_, and other well-known catalysts [56–59]. In order to predict the voltage range of OH^−^ filling, we first calculate the free energy of ∗OH adsorption in LNO with different ∗OH coverages (25%~100%) (Figure S29), and the result shows that the voltage range of ∗OH adsorption is 0.40 V~-0.78 V (vs. RHE) in pH 14 (Figure 3(e)). In cyclic voltammetry measurement, the significant oxidation peak at around 0.83 V (vs. RHE) is half chemisorption of ∗OH in a previous study [59]; thus, we marked this as the hydroxyl adsorption peak of LNO. The initial peak potential of Fe-LNO is lower than that of LNO, which proves that FeOOH can accelerate the adsorption of hydroxyl. Furthermore, the peak area of Fe-LNO is about two times higher than that of LNO, which also indicates the enhancement of adsorption capacity. To further prove the adsorption of hydroxyl within this voltage range, we also studied the signal of M-OH bond during 1.23 V (vs. RHE) by in situ Raman spectroscopy (Figure 3(f)). The peak at around 3270 cm^−1^ can be attributed to the O-H vibration peak of water, and that at 3468 cm^−1^ can be attributed to the H_2_O vibration [60]. Obviously, both of the peaks have no shift. However, compared with that of LNO, the M-OH peak of Fe-LNO shifts from 3599 cm^−1^ to 3610 cm^−1^ [61–63], and the positive shift of M-OH indicates that the vibration of M-OH is significantly enhanced, which is conducive to hydroxyl adsorption, consistent with DFT calculations. This further confirms that FeOOH can significantly promote the adsorption of hydroxyl of LNO.

The in situ Raman spectra of the catalysts in low frequency during OER are shown in Figure 3(g). For LNO, the peaks at around 470 cm^−1^ and 560 cm^−1^ are seen, and they are typical vibration peaks of Ni-O in NiOOH during OER, and this result is the same as previous reports [44, 64]. However, few of the above peaks are found in Fe-LNO, especially the peak at 570 cm^−1^; a significant peak of Fe-doped NiOOH is not seen either. The above results confirmed that the FeOOH can stabilize the structure of LNO by promoting hydroxyl adsorption, which is consistent with the time-dependent ICP results.

### 2.4. The Evidences of Weakened Ni-O Bond by FeOOH

**Figure 4:**
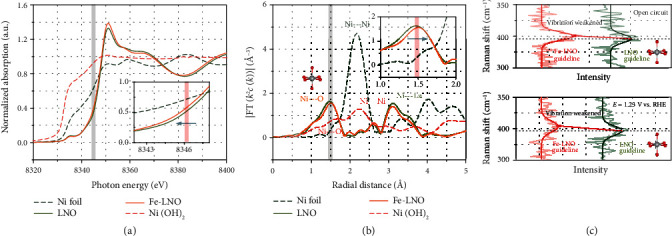
Evidences of weakened Ni-O bond. (a) Normalized nickel K-edge XANES spectra. (b) EXAFS *k*^2^*χ*(*k*) Fourier transform (FT) spectra of LNO and Fe-LNO with nickel foil as a reference. (c) In situ Raman spectra of Fe-LNO and LNO in open circuit and 1.23 V vs. RHE.

The weakened Ni-O bond in the Fe-LNO sample is demonstrated by in situ Raman spectrum and X-ray absorption fine-structure spectroscopy (XAFS). As shown in Figure 4(c), the Raman peak at around 400 cm^−1^ is a typical e_g_ vibration peak of LNO [65], and it can be assigned to the strength of Ni-O bond in NiO_5_ octahedra. The strength of Ni-O was increased in KOH solution, and this can be attributed to the anionic adsorption of the nickel layer on the surface [66]. We find that the e_g_ vibration peak of Fe-LNO samples shifted negatively, especially at 1.23 V vs. RHE, indicating that the FeOOH can weaken Ni-O bond of LNO. Furthermore, the K-edge XANES spectrum of nickel (Figure 4(a)) shows a slight shift toward lower energy, and this suggests the reduction state of Ni in Fe-LNO [67]. The K-edge XANES spectrum of Fe in Figure S32 shows a narrowed white-line peak, suggesting higher oxidation of Fe and the electron transport from FeOOH to LNO, in good agreement with the DFT calculations as shown in Figure S28b. The Ni K-edge extended XAFS (EXAFS) *k*^2^*χ*(*R*) spectra of LNO and Fe-LNO are shown in Figure 4(b), and there are two significant peaks at near 1.5 Å and 3.1 Å, which can be assigned to Ni-O peak and Ni-La peak, respectively [18]. The intensity of Ni-La peak of Fe-LNO decreases slightly compared with that of LNO, and there is no obvious position shift; however, the Ni-O peak of Fe-LNO is positively shifted compared with that of LNO, which further indicates that FeOOH can weaken the Ni-O bond of LNO.

### 2.5. The Evidences of LOER Promoted by FeOOH

**Figure 5:**
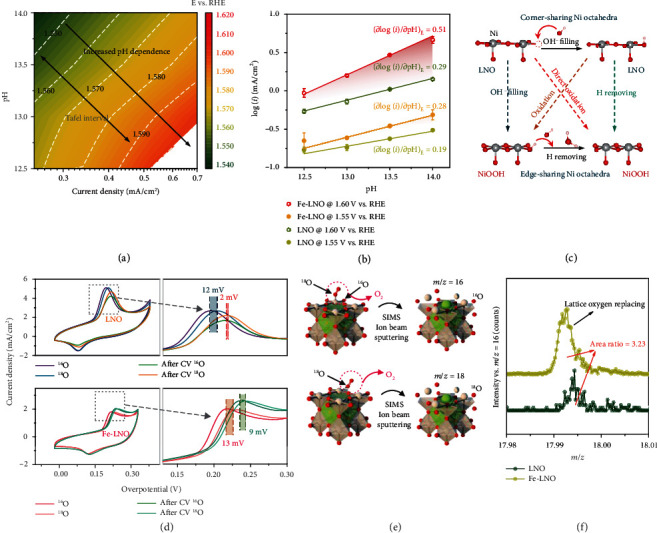
Evidences of LOER in Fe-LNO and LNO. (a) The rainbow map contains current density, voltage, and pH of LNO. (b) Specific OER catalytic activity at 1.55 V and 1.60 V versus RHE as a function of pH. (c) Two oxidation mechanisms of LNO and NiOOH. (d) Cyclic voltammograms in 0.1 mol/l Li^18^OH and LiOH of catalysts. (e) Illustration of the mechanism of ^18^O labeled TOF-SIMS. (f) The ^18^O signal in LNO and Fe-LNO (the intensity has been normalized by ^16^O signal).

OER electrocatalytic activity with pH dependence indicates the existence of NCPET step and confirms the presence of decoupled proton-electron transfer step. The previous studies have found that the LOER (especially in perovskite) shows significant pH dependence [11, 42]. In pH dependence tests, it is important to have a proper Tafel range to prevent the effect of electrolyte mass transfer efficiency [68]. We firstly plot the rainbow map including current density, voltage, and pH as shown in Figure 5(a). It is found that the pH dependence increased significantly after 1.60 V vs. RHE. Thus, the final Tafel range of LNO is locked in 1.53 V~1.60 V vs. RHE, which is consistent with previous reports [11, 36]. The oxide peak of Fe-LNO shows positive shift at 1.51 V~1.55 V (vs. RHE). In order to improve accuracy, we finally choose two potentials of 1.55 and 1.60 V (vs. RHE) to compare pH dependence of Fe-LNO and LNO as shown in Figure 5(b). Obviously, the pH dependence of Fe-LNO increases a factor of 1.5 compared with LNO, and this well confirms that the promotion of hydroxyl adsorption by FeOOH is an effective way to promote the LOER of perovskite. In addition, the specific OER catalytic activity (current normalized by geometry area) at 1.60 V (vs. RHE) as a function of pH is shown in Figure S34, and the same conclusion can be drawn.

To further confirm that FeOOH promotes LOER of LNO, the ^18^OH^−^ oxidation process in OER was detected by ^18^O labeling experiment. Figure 5(d) shows the 1^st^ and 50^th^ cyclic voltammograms (CVs) of LNO and Fe-LNO in 0.1 M Li^18^OH and 0.1 M Li^16^OH solutions. The 1^st^ CVs of LNO and Fe-LNO both show an obvious positive shift of oxidation peak in 0.1 M Li^18^OH solution compared with that in 0.1 M Li^16^OH solution. This phenomenon almost disappears for the 50^th^ CVs of LNO in 0.1 M Li^18^OH and 0.1 M Li^16^OH solutions, but it still exists for the Fe-LNO sample. To elucidate the different situations of LNO and Fe-LNO, we summarized two mechanisms of LNO and NiOOH as shown in Figure 5(c) [69]. As we all know, during the oxidation, the LNO undergoes OH^−^ oxidation step in O vacancy and H removing step [70], so the positive shift of oxidation peak in 0.1 M Li^18^OH solution compared with that in 0.1 M Li^16^OH solution indicates that the OH^−^ filling step is greatly influenced by isotope effect. After 50 cycles, the LNO becomes insensitive to ^18^OH^−^ because of the structure change; however, Fe-LNO still is sensitive to ^18^OH^−^ after 50 cycles, further indicating that FeOOH can enhance the LOER of LNO by the promotion of hydroxyl adsorption and prevention of the structure change of LNO from corner sharing to edge sharing, which is consistent with the results of in situ Raman spectroscopy. Moreover, we designed ^18^O isotope labeling mass spectrometry to confirm the existence of ^18^O in LNO and Fe-LNO during OER as shown in Figures 5(e) and 5(f). The significant peak of ^18^O in both LNO and Fe-LNO shows that the lattice oxygen exchange occurred during OER, which is consistent with the pH dependence test and ^18^O labeling test. As shown in Figure 5(f), the peak area of Fe-LNO is 3 times higher than that of LNO, which also confirmed FeOOH nanoclusters for optimizing the LOER of LNO.

### 2.6. Superior Electrochemical Performance of Fe-LNO

**Figure 6:**
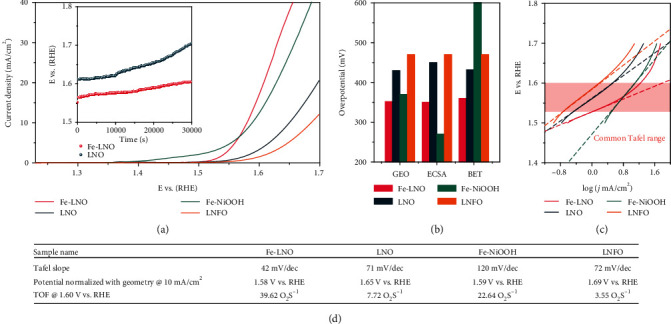
Electrochemical performance characterizations. (a) Oxygen evolution results and catalytic activities for LNO, Fe-LNO, Fe-NiOOH, and LNFO, activities measured in 1 M KOH at 10 mV s^−1^ and the chronopotentiometry of LNO and Fe-LNO measured at 10 mA cm^−2^. (b) The GEO, ECSA, and BET normalized overpotentials of various catalysts. (c) Tafel plots of the specific activity of each catalyst. (d) The Tafel slope, overpotential with geometry, and TOF of catalysts.

The catalytic activity and stability of Fe-LNO catalysts were assessed in the solution of 1.0 M KOH by cyclic voltammetry and chronopotentiometric at 10 mA/cm^2^ (current is normalized to geometric surface area of electrode) with a procedure similar to benchmarking studies for electrocatalysts [71]. In order to highlight the advantages of Fe-LNO, here, the catalytic performance of Fe-LNO catalysts was compared with those of LNO, Fe-NiOOH, and La_2_NiFeO_6_ (LNFO).  Figure 6(a) shows the chronopotentiometric and OER polarization curves of the series of catalysts. The Fe-NiOOH catalyst shows high OER catalytic activity with an overpotential of 360 mV at 10 mA cm^−2^ without IR correction. The LNO catalyst exhibits an overpotential of 420 mV at 10 mA cm^−2^, which is consistent with the earlier reports [72, 73]. However, the LNFO sample shows a poor performance compared with Fe-LNO, and it needs 460 mV to reach 10 mA cm^−2^. The above results show that the LNFO still cannot enhance the catalyst performance by doping Fe into LNO crystalline, which is also reported in a previous work [74]. Among the above various catalysts, the Fe-LNO showed the highest OER catalytic activity with the lowest overpotential of 350 mV to reach 10 mA cm^−2^, especially in high voltage range, as shown in Figure 6(a). The above results confirm that FeOOH can activate LOER of LNO by promoting hydroxyl adsorption, showing 5 times increase at 1.60 V (vs. RHE) compared with LNO. Furthermore, the effect of deposition time of FeOOH on LNO for 1~20 min on OER catalytic performance was investigated as shown in Figure S35a, which shows that the Fe-LNO owns the highest catalytic activity when the deposition time is 5 min. The stability tests of LNO and Fe-LNO are shown in the inset in Figure 6(a), which shows that the Fe-LNO owns much higher stability than LNO. So, the decoration of FeOOH can significantly improve the stability of LNO. In addition, we studied other similar perovskites modified by metal hydroxy oxides, such as CoOOH-LNO, MnOOH-LNO, and FeOOH-(Sr)LaCoO_3_, and their catalytic performance for OER also can be obviously improved as shown in Figure S41–S44. Therefore, the above results show that the decoration of metal hydroxy oxide is a universal method to improve the electrocatalytic performance of perovskite for OER [73, 75].

To evaluate the intrinsic performance, the electrochemically active surface area (ECSA) and Brunauer-Emmett-Teller surface area (BET) normalized LSVs of various catalysts were measured as shown in Figure S45, which shows that the Fe-LNO owns the highest BET normalized OER activity and Fe-NiOOH owns the highest ECSA normalized OER activity. The geometry area (GEO), ECSA, and BET normalized overpotentials of various catalysts are shown in Figure 6(b), and the LNFO is utilized as a reference catalyst with an overpotential of 460 mV. The Fe-LNO owns the lowest GEO and BET normalized overpotentials, and Fe-NiOOH owns the lowest ECSA normalized overpotentials. The above results show that the decoration of FeOOH did not enhance the ECSA of LNO but slightly decreased (the double-layer capacitance decreases from 2.98 to 1.83 mF cm^−2^), so the FeOOH cannot increase the active sites of LNO, and this is consistent with previous works [43, 76]. The Fe-NiOOH exhibits the lowest ECSA area (Figure S48b), which is attributed to the different mechanisms of double-layer capacitance between metal oxyhydroxide and oxide [77]. The BET normalized OER activity of Fe-LNO is almost the same as GEO and ECSA normalized OER activities. But Fe-NiOOH showed the poorest BET normalized OER activity, and this can be attributed to the maximum specific surface area of Fe-NiOOH, which was nearly 20 times than that of LNO (9.34 m^2^/g). So, the above results show that the catalytic performance of the Fe-LNO catalyst is much better than other catalysts, which shows that the intrinsic activity of perovskite can be effectively improved by the strategy of promoting hydroxyl adsorption.

The Tafel slope represents the OER kinetics of the catalyst. The Fe-LNO exhibits the smallest Tafel slope of 42 mV/dec as shown in Figure 6(c), which is much smaller than those of LNO and LNFO (71 mV/dec and 72 mV/dec), especially that of Fe-NiOOH (120 mV/dec). So, the Fe-LNO shows the excellent OER kinetic. Finally, we compared the turnover frequencies (TOFs) of LNO, Fe-LNO, Fe-NiOOH, and LNFO catalysts as listed in Figure 6(d), and Fe-LNO owns the biggest TOF value, further confirming that high OER catalytic activity of Fe-LNO is attributed to the promotion of hydroxyl adsorption that is an effective strategy to optimize OER of perovskite.

## 3. Conclusions

In summary, we have proposed an effective and feasible strategy to directly optimize the LOER of perovskite by the decoration of iron oxyhydroxide on the surface of perovskite. The designed Fe-LNO catalyst exhibited superior catalytic performance with five times OER catalytic activity enhancement and excellent long-term durability compared with the original LNO perovskite. Combining the structural characterization and DFT theoretical calculations, we well demonstrated that the FeOOH could strongly affect the coordination modes of anions and accelerate the hydroxyl adsorption step of LOER for LNO. Furthermore, the experimental results and DFT calculations both proved that the decoration of FeOOH could realize the positive shift of the O 2p band of LNO and accordingly could obviously decrease the energy barrier of LOM. The strategy of iron oxyhydroxide modification will open a surface engineering route to promote and optimize the LOER of perovskite and other metal oxides.

## 4. Materials and Methods

### 4.1. General

All chemicals were used as they were received from manufacturers. Lanthanum(III) nitrate hexahydrate (AR) and ferrous sulfate heptahydrate (AR) were purchased from Macklin. Nickel(II) nitrate hexahydrate (AR), iron(III) nitrate nonahydrate (AR), and potassium hydroxide (metal basis 99.999%) were purchased from Sigma-Aldrich. Citric acid monohydrate (AR) and absolute 200 proof ethanol were purchased from LookChem. 5 wt% Nafion solution in lower alcohols was obtained from Sigma-Aldrich. Millipore high-purity water (DI water, 18 M*Ω*) was used in this study.

### 4.2. Catalyst Synthesis

LaNiO_3_ (LNO) powder was synthesized by a Pechini method [14], followed by crystallization and annealing. Typically, A and B site nitrate salts with stoichiometric ratios were dissolved in water to form a solution, and the total concentration of metal salts was 0.2 M. Then, citric acid was added with a concentration of 0.2 M. The solution was stirred at 500 rpm for 1 h to ensure complete chelation of the metal cations. The clarified solution was dried at 120°C for 2 h to obtain colloid-like substances and then transfered to a tubular furnace. The heating rate was 1°C min^−1^, and the gel was combusted on a hot plate at 400°C to form mixed metal oxide precursor particles. This step was to avoid possible explosions from rapid evolution of gasses upon combustion. Finally, the precursor particles were crystallized at 800°C for 4 h, slowly cooling to room temperature.

La_2_NiFeO_6_ (LNFO) powder was synthesized by the same method as that of LNO. XRD of LNFO is shown in Figure S9b, and the negative shift of the peak of LNFO confirmed the success of Fe doping, which is consistent with previous reports [78, 79].

FeOOH-LaNiO_3_ (Fe-LNO) was obtained by a simple deposition way. Typically, 0.246 g of LNO sample was immersed into a 100 ml FeSO_4_·6H_2_O (2.78 g) aqueous solution, then was stirred under RT for 5 min. The pH of solution must be held around 4 to prevent LNO from being etched. Then, the above solution was centrifuged for three times, and the obtained solid powders were further oxidized at 70°C in air to synthesize Fe-LNO.

NiOOH was obtained by a chemical coprecipitation way. The Ni(NO_3_)_2_ and KOH in stoichiometric ratios were dissolved in water to form a solution. Then, the above solution was centrifuged for three times to synthesize NiOOH powder. The decoration of FeOOH on NiOOH (Fe-NiOOH) was synthesized by the reaction of Ni(NO_3_)_2_ and KOH, and the obtained Ni(OH)_2_ was the typical *β* phase, with high specific area of 170 m^2^/g, as shown in Figure S5. The obtained *β*-Ni(OH)_2_ was then further oxidized to NiOOH by cycle voltammetry. Finally, the deposition of FeOOH has the same procedure as that of Fe-LNO.

### 4.3. Material Characterization

The surface morphology of catalysts was characterized by a Zeiss Sigma field emission scanning electron microscopy (FE-SEM, JSM-6330F). The structure of samples was measured with transmission electron microscopy (TEM), JEM-2010HR, and high-resolution TEM (HRTEM, 200 kV or 300 kV), and the fast fourier transform (FFT) images were obtained by Digital Micrograph software. The specific surface area was characterized by nitrogen adsorption-desorption measurements (Micromertics ASAP 2020 M) using the Brunauer-Emmett-Teller (BET) method. 2.0 g of the sample was pretreated at 110°C for at least 2 h before N_2_ physisorption measurements at 77 K. X-ray photoelectron spectroscopy (XPS) was performed by an ESCA Lab250 X-ray photoelectron spectrometer. All XPS spectra were corrected using the C 1s line at 284.8 eV, and the curve fitting and background subtraction were accomplished. X-ray absorption near edge structure (XANES) measurements of Ni and Fe K-edge were conducted on the 06ID superconducting wiggler sourced hard X-ray microanalysis beamline Beijing Synchrotron Radiation Facility. Each sample spectrum was collected in a transmission mode for 2 comparison and monochromatic energy calibration. The catalyst structure was examined by performing X-ray diffraction with the Bruker-D8 advance diffractometer, and the measurements were performed at 298 K in ambient conditions while the instrument operated at 40 kV and 15 mA using Cu K*α* radiation (1.54 Å wavelength). Catalyst powder was exposed to ambient air and scanned over 20~80° in 0.02° increments with a step time of 0.2 s. The LNO was scanned over 10~105° in 0.02 increments with a step time of 95 s to get high-quality PXRD pattern for further Rietveld refinement. The Rietveld refinement was performed with the FullProf refinement program [80]. The crystal structure of the R3̅c space group was used as a starting model of LNO [81].

### 4.4. Electrochemical Characterization

The catalyst inks were obtained by mixing 3 mg catalyst powders with 1 ml NaOH solution (1.0 M) and then were neutralized with 0.05 wt% Nafion solution. The catalyst inks must be bath sonicated for at least half an hour before use. Ten microliters of catalyst inks was drop cast onto 5 mm glassy carbon electrode (GCE) and dried with infrared lamp. Before use, the GCE was polished using 0.05 *μ*m alumina powder and washed with fresh high-purity water and ethanol mixed solution; then, it was sonicated in a fresh solution of water, and finally, it was dried with infrared lamp. The above steps were used to clean all GCEs before drop casting. The pH dependence test and other electrochemical characterizations were detailed in supporting information (Figure S38).

Electrochemical performance of catalyst was assessed on a CHI-760E workstation at room temperature. The electrolyzer cell of three electrodes was used for all experiments. The reference electrode was a KCl saturated calomel Hg/Hg_2_Cl_2_ electrode, the Pt plate electrode served as the counter electrode, and GCE coated with catalysts was used as the working electrode. All potentials were measured vs. Hg/Hg_2_Cl_2_, but they were used versus reversible hydrogen electrode (RHE) and were determined by *U*_RHE_ = *U*_SCE_ + 0.241 + 0.059∗lg(pH).

Cycle voltammetry of the OH^−^ adsorption test was performed from 0.23 V to 1.57 V vs. RHE at a scan rate of 10 mV s^−1^, and the OER polarization curve was measured from 1.23 V to 1.73 V vs. RHE at the same rate including the pH dependence test. All the curves were using the 10^th^ cycle of the cycle voltammetry data and also compared the performance of the 1^st^ cycle. The current density at 10 mA cm^−1^ was used for the comparison of OER catalytic activities; data presented in this report are the average of at least three tests on fresh electrodes. The ^18^O labeled LiOH was synthesized by hydrogen substitution of heavy oxygen water (H_2_^18^O, Macklin); the process proceeds slowly in an ice bath and accesses the N_2_ during reaction to prevent the formation of Li_2_O. The obtained Li^18^OH was dissolved in high-purity water (H_2_^16^O) to reach pH of 13. The ^16^O labeled LiOH was synthesized by the same way to compare the barrier of oxidation. Cycle voltammetry was performed from 1.23 V to 1.61 V vs. RHE for LNO and 1.23 V to 1.56 V vs. RHE for Fe-LNO at a scan rate of 10 mV s^−1^ to prevent intense oxidation. The further cycle voltammetry for oxidation was performed from 1.27 V to 1.87 V vs. RHE for 50 cycles to detect the structure change of LNO.

### 4.5. *In Situ* Raman Spectroscopy

All the in situ Raman spectroscopy experiments were carried out at an inVia confocal Raman microscope, Renishaw. The water immersion objective is 50x and wrapped in a Teflon film (0.025 mm in thickness) to avoid direct contact between lens and electrolyte (Figure S22). The laser power was 532 nm with 0.5% power (prevent the Teflon film from being oxidized) at grating of 1800 mm^−1^. The silicon peak of 520 cm^−1^ is correct before testing to ensure the instrument accuracy. All the in situ electrochemical characterizations were using a three-port electrolyzer, with the same reference with previous characterizations. The electrolyte was using the metal basis KOH of 1.0 M; the open circuit represents to the powder immersed in KOH while the 1.23 V and 1.73 V vs. RHE were used to detect the OH^−^ filling step and material changes during OER, respectively.

### 4.6. OH^−^ Adsorption pH Test

The OH^−^ adsorption pH test was performed with a pH meter (PHS-2F). 1 M metal basis KOH was used for detecting. The solution of 10 ml KOH contains 100 mg total weight of catalysts, and the obtained catalyst ink was sonicated 5 min until dispersible, then precipitate to test the pH of supernatant. The mole amount of OH^−^ adsorption was calculated by converted OH^−^ concentration. The initial pH value for all the samples is 13.28, while the end pH values are 13.26, 13.17, and 12.96 for LNO, Fe-LNO, and FeOOH, respectively. The formula is shown as follows:

(1) $$\begin{array}{c} \frac{\left(10^{14-{\text{pH}}_{\text{initial}}}-10^{14-{\text{pH}}_{\text{final}}}\right)*V_{\text{electrolyte}}}{m_{\text{sample}}*\left(1/M_{\text{sample}}\right)}, \end{array}$$

where *V* denotes the volume of the electrolyte and *m* and *M* denote the mass and relative molecular mass of the samples, respectively.

### 4.7. Time-Dependent ICP-AES and ICP-MS Characterizations

The characterizations of time-dependent inductively coupled plasma-atomic emission spectrometry (ICP-AES) and inductively coupled plasma-mass spectrometer (ICP-MS) were carried out with iCAP Qc and IRIS(HR), TJA, respectively. The chronopotentiometry was measured at 10 mA/cm^2^ in 1 M KOH solution of 10 ml. 1 ml of electrolyte solution for OER was taken to dilute to 10 ml with 1 mol l^−1^ HNO_3_ for the ICP test.

### 4.8. Secondary Ion Mass Spectroscopy (SIMS) Characterization

SIMS was conducted to determine the ^18^O and ^16^O isotopes in catalysts using a TOF-SIMS ion tof Gmhb 5 device. Firstly, the catalysts were dispersed on Ti foil (5 mm∗5 mm) and were activated using cyclic voltammetry (CV) from 0.80 to 1.60 V (vs. RHE) at a scan rate of 10 mV s^−1^ for 50 cycles in 1 M Li^18^OH aqueous solution. Then, rinsing with ^16^O water, the catalysts were finally heated at 65°C for 6 h to remove adsorbed H_2_^18^O.

### 4.9. Density Function Theory Calculations and Surface Models

Spin-polarized density functional theory (DFT) calculations were performed in the plane wave and ultrasoft pseudopotential as implemented in Quantum ESPRESSO [82]. The adsorption energies were calculated using the Grimme-D^3^ vdw-correction with Perdew-Burke-Ernzerhof (PBE) exchange functional correction [83]. The effective *U*‐*J* terms, from linear response theory [84], were 3.5 and 6.6 for Fe and Ni, respectively. The kinetic energy cutoffs of 25 Ry and 225 Ry were chosen for the wave functions and augmented charge densities. All the atomic structures for the models were fully relaxed with self-consistency criteria of 10^−5^ Ry, and all atomic coordinates were converged to within 10^−3^ Ry/bohr for maximal components of forces. The occupancy of the one-electron states was calculated using an electronic temperature of *k*_*B*_*T* = 0.01 Ry for surfaces and 10^−3^ Ry for molecules in vacuum. All energies were extrapolated to *T* = 0 K. The vacuum slab of 12 Å was used for surface isolation to prevent interaction between two surfaces. All the atoms were relaxed to simulate a bulk structure, and all atoms were fixed except the top two layers in the slab system. Reciprocal space was sampled by the Γ-centered Monkhorst-Pack scheme with (lattice parameters × *k*) ~ 30 to compare the energy differences. Due to the complex structure of the R3̅c space group, we use the cubic phase for all calculations. The cell parameter of LNO is *a* = *b* = *c* = 3.86 Å, which agrees well with experiment [54]. Fe-LNO was built by supercell matching; the lattice mismatching error was less than 8%. The calculation of the OER pathway was detailed in supporting information (Figures S18–S29).

## Supplementary Materials

Supplementary Materials Figure S1: pH values of the different solutions: (1) H_2_O, (2) Fe(NO_3_)_3_ solution, (3) FeCl_3_ solution, and (4) FeSO_4_ solution. All salt solutions have the same mass: 100 mg Fe salt dissolved in 10 ml deionized water. Figure S2: SEM images of LNO. The specific surface area is 9.34 m^2^ g^−1^. Figure S3: SEM images of Fe-LNO. The specific surface area is 14.77 m^2^ g^−1^. Figure S4: SEM images of La_2_NiFeO_6_. The specific surface area is 10.74 m^2^ g^−1^. Figure S5: SEM images of NiOOH. The specific surface area is 170.58 m^2^ g^−1^. Figure S6: SEM images of NiO. The specific surface area is 3.23 m^2^ g^−1^. Figure S7: HRTEM images of LNO, and the lattice constant was 0.38 nm, close to the experimental value of 3.86 Å. Figure S8: Rietveld refined XRD patterns of LNO. The rhombohedral structure (space group: R3c) of LNO with a lattice constant of *a* = *b* = 5.457 Å, *c* = 13.180 Å. Figure S9: XRD patterns of Fe-LNO (a), La_2_NiFeO_6_ (b), NiO (c), and NiOOH (d). Figure S10: Raman spectra of LNO (a) and Fe-LNO (b). The peak at around 560 cm^−1^ is closest in shift to *α*-FeOOH. Figure S11: (a) in situ Raman spectroscopic device photograph. The in situ Raman spectra of (b) LNO and (c) Fe-LNO at 1.73 V vs. RHE. Figure S12: high-resolution XPS spectra of (a) Ni 2p, La 3d, and (b) O 1s of LNO and Fe-LNO. Figure S13: the XPS survey spectra of the as-synthesized LNO and Fe-LNO. Figure S14: (a) high-resolution O 1s XPS spectra of FeOOH. The ratios of peak areas of H_2_O : OH- : O_2_^2-^ is about 3.5 : 3.5 : 1, which is higher than LNO but lower than Fe-LNO, proving that hydroxyl adsorption does not only come from FeOOH; (b) high-resolution Fe 2p XPS spectra of FeOOH and Fe-LNO. Figure S15: projected density of states of Ni (3d) orbitals in bulk NiO_5_ (a) and surface NiO_5_ (b). All calculation results were normalized with the Fermi level. Figure S16: projected density of states of O (2p) orbitals in bulk NiO_5_ (a) and surface NiO_5_ (b). All calculation results were normalized with the Fermi level. Figure S17: integral curve of Ni 3d density of states in bulk NiO_5_ (a) and surface NiO_5_ (b). Figure S18: the proposed two OER mechanisms of LNO with the same starting point (Ni-∗OH). The dashed box represents the most important step in the adsorbate evolution mechanism and lattice oxygen participation mechanism. Figure S19: the free energies of LNO with the same starting point (the dashed box represents the most important step in the adsorbate evolution mechanism and lattice oxygen participation mechanism). Figure S20: Four concerted proton-coupled electron transfer mechanisms for OER occurring at Ni site (Ni) (in red) and possible sequential proton-electron transfers along the path, constructed with the assumption that the charge of the reaction intermediates does not exceed one electron. Figure S21: oxygen evolution reaction pathway of LNO, slab model (a), M-OH (b), M-O (c), M-OOH (d), and M-OO (e). All the structures are calculated in the same parameter. Figure S22: lattice oxygen participated oxygen evolution reaction pathway of LNO, slab model with lattice oxygen vacancy (a), M(Ov)-OH+VO (b), M(Ov)-∗OH-∗OH (c), M(Ov)-∗OH (d), and M(Ov)-∗OO (e). All the structures are calculated in the same parameter. Figure S23: bulk models of LNO with top view (a) and side view (b). (The green, red, and gray balls represent La, O, and Ni atoms, respectively. The lattice constant of unit cell is *a* = *b* = *c* = 3.857 Å.) Figure S24: bulk models of FeOOH with top view (a) and side view (b). (The brown and red balls represent Fe and O atoms, respectively.) Figure S25: slab models of LNO with side view (a) and perspective view (b). The vacuum layer was 12 Å. The top layer of NiO_5_ is relax while other inner layer is fixed. Figure S26: slab models of Fe-LNO with side view (a) and left view (b). The vacuum layer was 12 Å. The whole FeOOH layer and the top layer of NiO5 are relax while other inner layers are fixed. The calculation of ∗OH adsorption energy is simulated by bulk catalysis. Figure S27: DFT calculated electronic structures for Figure 1. (a) The charge distribution of Fe-LNO; (b) the charge density difference on Fe-LNO: *ρ* = *ρ*[Fe‐LNO] + *ρ*(FeOOH)‐*ρ*[LaNiO_3_]; yellow and cyan isosurface represents electron accumulation and electron depletion, respectively. Figure S28: (a) top view of the charge density difference on Fe-LNO. (b) Isosurface 2D view of the surface NiO_5_. Figure S29: the free energy of ∗OH adsorption in LNO with different ∗OH coverages, 25% (a)~100% (b). The related change of free energy of reaction (c, d). The coverage of 100% is using a single unit cell to construct, while the 25% is using super cell with 2∗2 in the *xy* axis. Figure S30: the images of SIMS tests of LNO (top), Fe-LNO (middle), and FeOOH (bottom). Figure S31: the area ratio of Fe-LNO and LNO supported on FeOOH. Figure S32: normalized Fe K-edge XANES spectra and EXAFS *k*2*χ*(*k*) Fourier transform (FT) spectra of FeOOH and Fe-LNO with Fe foil as a reference. Figure S33: the soft-XAFS spectrum of Fe-LNO. Figure S34: specific OER catalytic activity (current normalized by geometry area) at the current density at 1.60 V (versus RHE) as a function of pH. Figure S35: the effect of different deposition times of Fe on catalytic performance and the ratio of Fe to Ni at different deposition times. The ratio is obtained by ICP-AES, and it can be found that the ratio of Fe to Ni is the best at 0.12. Figure S36: the OER activities of FeOOH-NiO and Fe-LNO normalized to the geometry area. NiO powder was synthesized using a Pechini method, followed by crystallization and annealing, as the same procedure of LNO. Figure S37: cyclic voltammetry stability tests of Fe-LNO (a) and LNO (b). A total of 50 cycles (1.27 V~1.62 V vs. RHE) at a scan rate of 10 mV s^−1^ in 1.0 M KOH. Figure S38: CV measurements of KOH solution with different pH values recorded on (a) Fe-LNO and (b) LNO at 10 mV s^–1^. All the KOH was used in metal basis 99.999%. The catalyst inks were mixed by 3 mg catalyst powder with 1 ml NaOH neutralized 0.05 wt% Nafion solution and bath sonicated for at least half an hour. Three microliters of catalyst ink was drop cast onto 3 mm glassy carbon electrodes (GCE). Figure S39: CV measurements from 0.03 M KOH (pH 12.5) to 1 M KOH (pH = 14) recorded at 10 mV s^–1^: (a) Fe-LNO; (b) LNO (all the KOH was used in metal basis 99.999%). Figure S40: the OER catalytic activities of catalysts in 1.0 M KOH solution saturated with O_2_ and N_2_: (a) LNO, (b) Fe-LNO. Figure S41: (a) the OER catalaytic activities of various samples (LaNiO_3_, CrO_*x*_H_*y*_‐LaNiO_3_, MnO_*x*_H_*y*_‐LaNiO_3_, FeO_*x*_H_*y*_‐LaNiO_3_, CoO_*x*_H_*y*_‐LaNiO_3_, NiO_*x*_H_*y*_‐LaNiO_3_, and CuO_*x*_H_*y*_‐LaNiO_3_) normalized to the geometry area at a scan rate of 5 mV s^−1^ in 1.0 M KOH. (b) The related Tafel curves of various samples. Figure S42: (a) the OER catalytic activities of samples LaCoO_3_ and FeOOH-LaCoO_3_ normalized to the geometry area at a scan rate of 5 mV s^−1^ in 1 M KOH; (b) the related Tafel curve of samples. Figure S43: (a) the OER catalytic activities of samples La_0.75_Sr_0.25_CoO_3_ and FeOOH-La_0.75_Sr_0.25_CoO_3_ normalized to the geometry area at a scan rate of 5 mV s^−1^ in 1.0 M KOH; (b) the related Tafel curve of samples. Figure S44: (a) the OER catalytic activities of samples La_0.5_Sr_0.5_CoO_3_ and FeOOH-La_0.5_Sr_0.5_CoO_3_ normalized to the geometry area at a scan rate of 5 mV s^−1^ in 1 M KOH; (b) the related Tafel curve of samples. Figure S45: the OER activities of samples Fe-LNO, La_2_NiFeO_6_, NiO, and NiOOH normalized to the BET surface area (a) and ECSA surface area (b). The conversion method is by dividing the geometric area directly by the corresponding ECSA and BET values. Figure S46: double-layer capacitance measurements for determining the electrochemically active surface area (ECSA) of LNO from cyclic voltammetry in 1.0 M KOH solution. (a) Typical cyclic voltammograms in the non-Faradaic region around 0.05 V relative to SCE at different scan rates (0.01, 0.02, 0.03, 0.04, and 0.05 V s^−1^); (b) the average of the cathodic and anodic charging current absolute values measured at 0.025 V (vs. SCE) plotted as a function of the scan rate. Figure S47: double-layer capacitance measurements for determining electrochemically active surface area (ECSA) of Fe-LNO from cyclic voltammetry in 1.0 M KOH solution. (a) Typical cyclic voltammograms in the non-Faradaic region around 0.05 V relative to SCE at different scan rates (0.01, 0.02, 0.03, 0.04, and 0.05 V s^−1^); (b) the average of the cathodic and anodic charging current absolute values measured at 0.025 V (vs. SCE) plotted as a function of the scan rate. Figure S48: double-layer capacitance measurements for determining electrochemically active surface area (ECSA) of Fe-NiOOH from cyclic voltammetry in 1.0 M KOH solution. (a) Typical cyclic voltammograms in the non-Faradaic region around 0.05 V relative to SCE at different scan rates (0.01, 0.02, 0.03, 0.04, and 0.05 V s^−1^); (b) the average of the cathodic and anodic charging current absolute values measured at 0.025 V (vs. SCE) plotted as a function of the scan rate. Figure S49: double-layer capacitance measurements for determining electrochemically active surface area (ECSA) of La_2_NiFeO_6_ from voltammetry in 1.0 M KOH solution. (a) Typical cyclic voltammograms in the non-Faradaic region around 0.05 V relative to SCE at different scan rates (0.01, 0.02, 0.03, 0.04, and 0.05 V s^−1^); (b) the average of the cathodic and anodic charging current absolute values measured at 0.025 V (vs. SCE) plotted as a function of the scan rate. Figure S50: XRD patterns of Fe-LNO after different cycle times.
