# Supplementary material for: Boosting Lattice Oxygen Oxidation of Perovskite to Efficiently Catalyze Oxygen Evolution Reaction by FeOOH Decoration
Source: Research (Wash D C). 2020 Jul 10;2020:6961578. doi: 10.34133/2020/6961578 (PMC7368968; doi:10.34133/2020/6961578)
Supplement: Supplementary Materials — Figure S1: pH values of the different solutions: (1) H2O, (2) Fe(NO3)3 solution, (3) FeCl3 solution, and (4) FeSO4 solution. All salt solutions have the same mass: 100 mg Fe salt dissolved in 10 ml deionized water. Figure S2: SEM images of LNO. The specific surface area is 9.34 m2 g−1. Figure S3: SEM images of Fe-LNO. The specific surface area is 14.77 m2 g−1. Figure S4: SEM images of La2NiFeO6. The specific surface area is 10.74 m2 g−1. Figure S5: SEM images of NiOOH. The specific surface area is 170.58 m2 g−1. Figure S6: SEM images of NiO. The specific surface area is 3.23 m2 g−1. Figure S7: HRTEM images of LNO, and the lattice constant was 0.38 nm, close to the experimental value of 3.86 Å. Figure S8: Rietveld refined XRD patterns of LNO. The rhombohedral structure (space group: R3c) of LNO with a lattice constant of a = b = 5.457 Å, c = 13.180 Å. Figure S9: XRD patterns of Fe-LNO (a), La2NiFeO6 (b), NiO (c), and NiOOH (d). Figure S10: Raman spectra of LNO (a) and Fe-LNO (b). The peak at around 560 cm−1 is closest in shift to α-FeOOH. Figure S11: (a) in situ Raman spectroscopic device photograph. The in situ Raman spectra of (b) LNO and (c) Fe-LNO at 1.73 V vs. RHE. Figure S12: high-resolution XPS spectra of (a) Ni 2p, La 3d, and (b) O 1s of LNO and Fe-LNO. Figure S13: the XPS survey spectra of the as-synthesized LNO and Fe-LNO. Figure S14: (a) high-resolution O 1s XPS spectra of FeOOH. The ratios of peak areas of H2O : OH- : O22- is about 3.5 : 3.5 : 1, which is higher than LNO but lower than Fe-LNO, proving that hydroxyl adsorption does not only come from FeOOH; (b) high-resolution Fe 2p XPS spectra of FeOOH and Fe-LNO. Figure S15: projected density of states of Ni (3d) orbitals in bulk NiO5 (a) and surface NiO5 (b). All calculation results were normalized with the Fermi level. Figure S16: projected density of states of O (2p) orbitals in bulk NiO5 (a) and surface NiO5 (b). All calculation results were normalized with the Fermi level. Figure S17: integral curve [file 6961578.f1.docx]

**Supporting Information**

**Boosting Lattice Oxygen Oxidation of Perovskite to Efficiently Catalyze Oxygen Evolution Reaction by FeOOH Decoration**

Jia-Wei Zhao^1^, Cheng-Fei Li^1^, Zi-Xiao Shi, Jie-Lun Guan and Gao-Ren Li*

*MOE Laboratory of Bioinorganic and Synthetic Chemistry, The Key Lab of Low-carbon Chemistry & Energy Conservation of Guangdong Province, School of Chemistry, Sun Yat-sen University, Guangzhou 510275, China*

**Free energy calculations**

The OER free energy profiles were derived using the same scheme utilized in previous studies^[1]^. The free energy change of any elementary step that involves the production of a proton-electron pair will decrease linearly with applied potential versus the RHE:

$$\Delta G=\Delta G^{o}-eU_{\mathrm{RHE}}$$

The theoretical overpotential at which the step becomes downhill may be calculated as:

$$\eta=\max_{i}(\Delta G_{i}^{o})/e-E^{o}$$

where $E^{o}$ is the equilibrium potential of the reaction (1.23 V in the case of OER) and *e* is the fundamental unit of charge. The adsorbates evolution mechanism (AEM) contains four elementary steps^[2]^, and each of these steps contains a single proton electron transfer. The free energy of each adsorbate is calculated at 0 V vs RHE by referencing to liquid water and hydrogen gas at standard conditions:

$$\Delta G_{\mathrm{OH}^{*}}=\Delta E_{\mathrm{OH}^{*}}-\mu_{H_{2}O\left( l \right)}^{o}+\frac{1}{2}\mu_{H_{2}\left( g \right)}^{o}+\Delta H_{\mathrm{OH}^{*}}+\Delta Z_{\mathrm{OH}^{*}}-T\Delta S_{\mathrm{OH}^{*}}$$

$$\Delta G_{O^{*}}=\Delta E_{O^{*}}-\mu_{H_{2}O\left( l \right)}^{o}+\mu_{H_{2}\left( g \right)}^{o}+\Delta H_{O^{*}}+\Delta Z_{O^{*}}-T\Delta S_{O^{*}}$$

$$\Delta G_{\mathrm{OOH}^{*}}=\Delta E_{\mathrm{OOH}^{*}}-2\mu_{H_{2}O\left( l \right)}^{o}+\frac{3}{2}\mu_{H_{2}\left( g \right)}^{o}+\Delta H_{\mathrm{OOH}^{*}}+\Delta Z_{\mathrm{OOH}^{*}}-T\Delta S_{\mathrm{OOH}^{*}}$$

where $\Delta E_{i}$ is the *i* chemisorption energy and $\Delta Z_{i}$, $\Delta H_{i}$ is the difference in zero-point energy between the adsorbed and enthalpy, $\Delta S_{i}$ is the gas phase entropy change of *i* adsorption. $\Delta E_{H}$ is computed by: $\Delta E_{H}=E_{slab+i}-E_{\mathrm{slab}}-\mu_{l}^{o}$, where $\mu_{l}^{o}$ can be definition by:

$$\mu_{H^{+}/e^{-}}^{o}=\mu_{H_{2}\left( g \right)}^{o}$$

$$\mu_{H_{2}O\left( l \right)}^{o}=\mu_{H_{2}O\left( l \right)}^{o}|_{p=0.035 bar}@T=300K$$

The correction calculated using the harmonic approximation for every adsorbate and surface, with typical values of +0.35 eV, +0.05 eV, +0.40 eV, -0.03 eV and +0.27 eV for HO*, O*, HOO*, OO* and V_O_ + HO*, respectively.

Final $\Delta G_{i^{*}}$ formula can be summarized by:

$$\Delta G_{1}=\Delta E_{\mathrm{OH}^{*}}+0.35 eV-eU$$

$$\Delta G_{2}=\Delta E_{O^{*}}-\Delta E_{\mathrm{OH}^{*}}-0.30 eV-eU$$

$$\Delta G_{3}=\Delta E_{\mathrm{OOH}^{*}}-\Delta E_{O^{*}}+0.35 eV-eU$$

$$\Delta G_{4}=\Delta E_{\mathrm{OO}^{*}}-\Delta E_{\mathrm{OOH}^{*}}-eU$$

It is worth mentioning that some structures can’t be normally optimized under different adsorption conditions. For example, $\mathrm{OOH}^{*}$ steps in the normal OER process on LaNiO_3_ surface will meet great optimization problems. Therefore, for these special cases, the linear relationship between the adsorbed species is predicted by using the previous reports of norskov et al ^[1]^. The formula is as follows:

$$\Delta E_{\mathrm{OO}^{*}}=0.64\Delta E_{O^{*}}+2.03 eV=1.64\Delta E_{\mathrm{OH}^{*}}+1.48 eV$$

Similar calculations are also conducted for the lattice oxygen mechanism (LOM)

**Turnover frequency calculations**

The turnover frequency calculations is performed with previous reports^[3]^, specifically:

$$TOF =\frac{\# Total oxygen turn overs / \mathrm{cm}^{2} geometric area}{\# Surface sites / \mathrm{cm}^{2} geometric area}$$

The total number of oxygen turn overs was calculated from the current density:

$$\# Total oxygen turn overs =j\frac{mA}{\mathrm{cm}^{2}}*\frac{1 Cs^{-1}}{1000 mA}*\frac{1 mol e^{-}}{96485.3 C}*\frac{1 mol O_{2}}{4 mol e^{-}}*\frac{6.022*{10}^{23}O_{2}}{1 mol O_{2}}=1.56*{10}^{15}O_{2} s^{-1}$$

Because the active sites of oxygen evolution reaction are not very clear, the main metal sites are used as catalytic sites. We use the surface sites per real surface area to calculate the surface sites.

$$\# Surface sites ={(\frac{active atoms in unit cell / unit cell}{Unit-cell volume / unit cell})}^{0.667}$$

**Supplementary Figures**


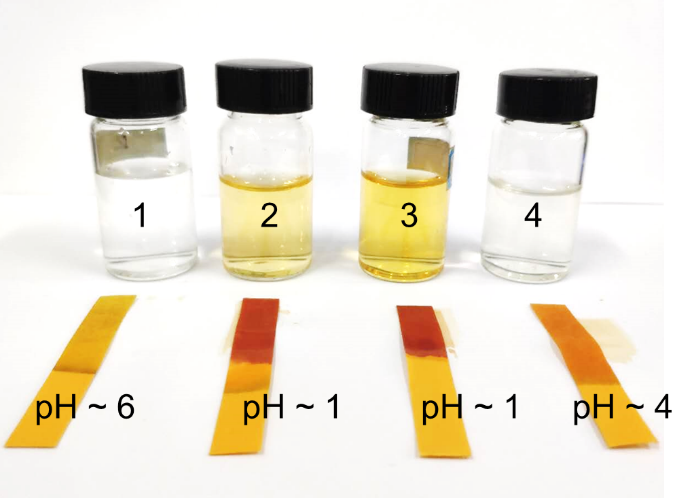


**Figure S1.** pH values of the different solutions: (1) H_2_O, (2) Fe(NO_3_)_3_ solution, (3) FeCl_3_ solution, and (4) FeSO_4_ solution. All salt solutions have the same mass: 100 mg Fe salt dissolved in 10 ml deionized water.


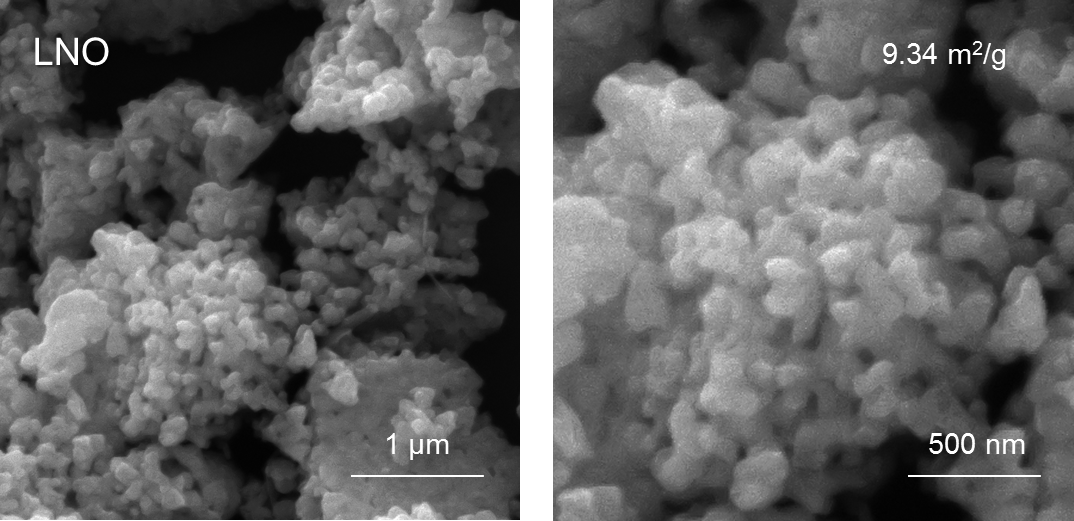


**Figure S2.** SEM images of LNO. The specific surface area is 9.34 m^2^ g^-1^.


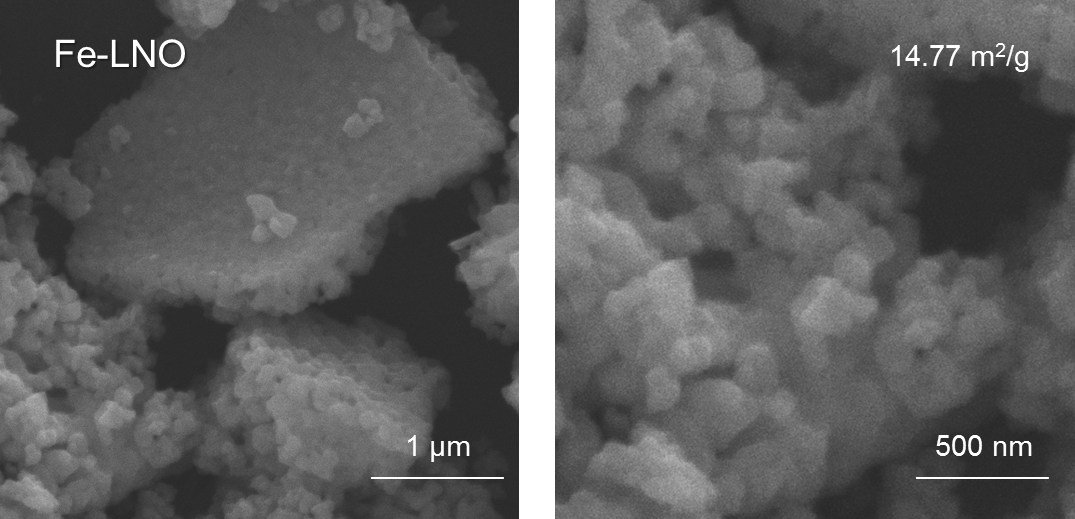


**Figure S3.** SEM images of Fe-LNO. The specific surface area is 14.77 m^2^ g^-1^.


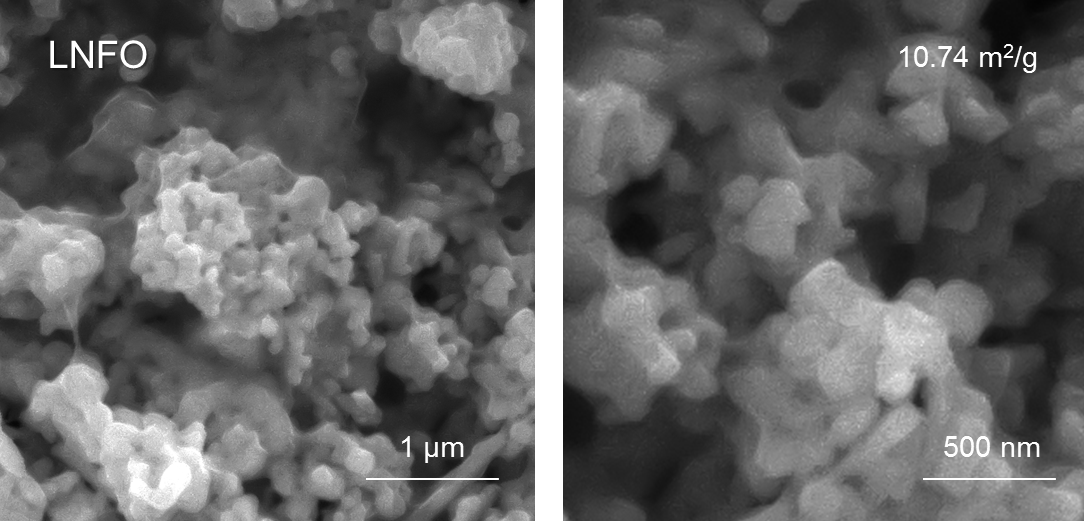


**Figure S4.** SEM images of LNFO. The specific surface area is 10.74 m^2^ g^-1^.


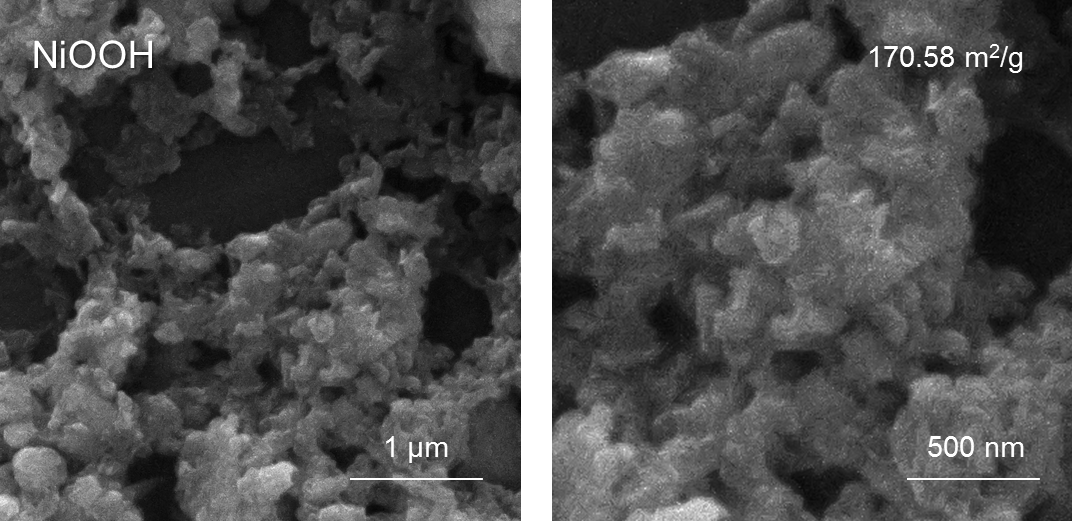


**Figure S5.** SEM images of NiOOH. The specific surface area is 170.58 m^2^ g^-1^.


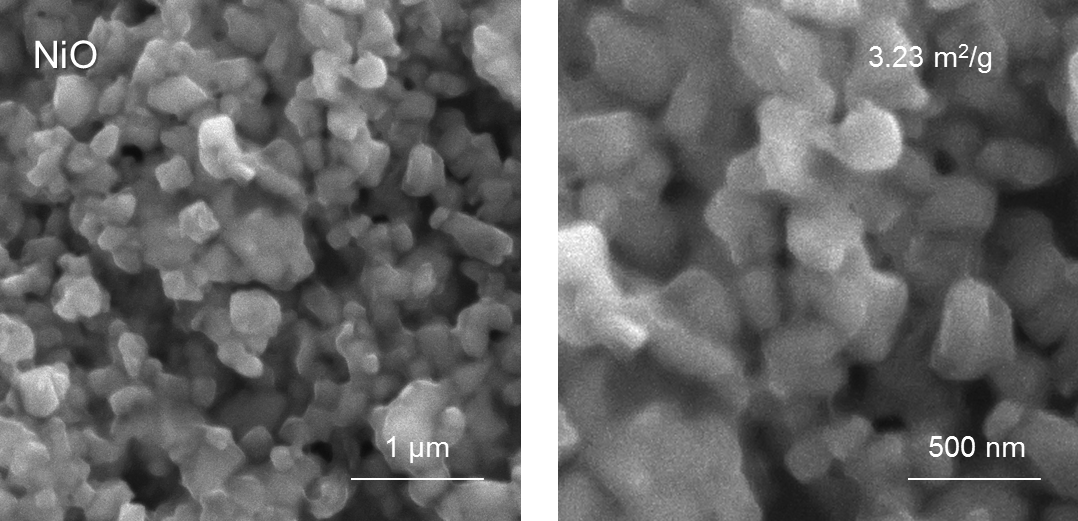


**Figure S6.** SEM images of NiO. The specific surface area is 3.23 m^2^ g^-1^.


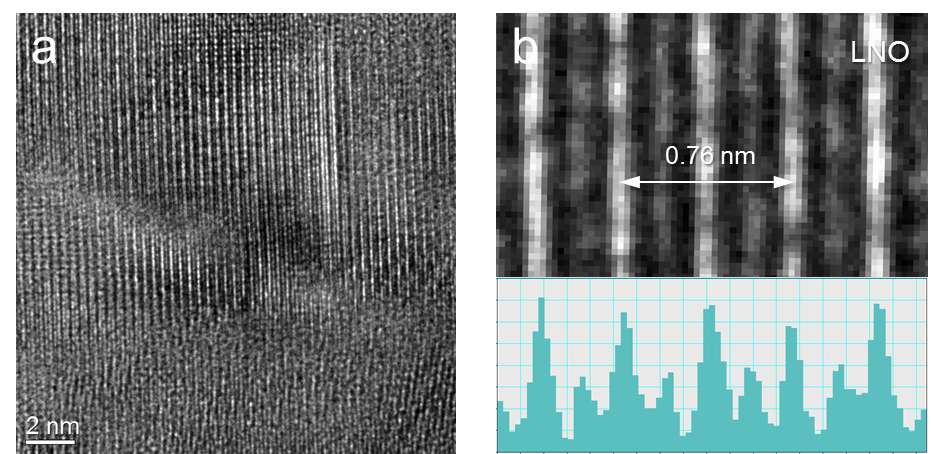


**Figure S7.** HRTEM images of LNO and the lattice constant was 0.38 nm, close to the experimental value of 3.86 Å.


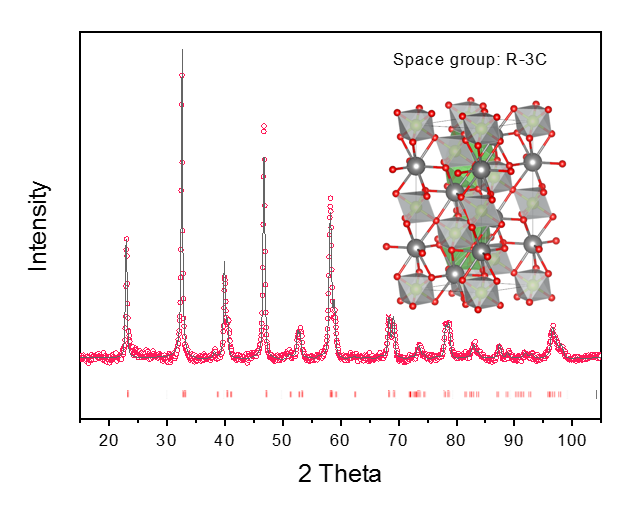


**Figure S8.** Rietveld refined XRD patterns of LNO. The rhombohedral structure (space group: R3c) of LNO with a lattice constant of a = b =5.457 Å, c = 13.180 Å.


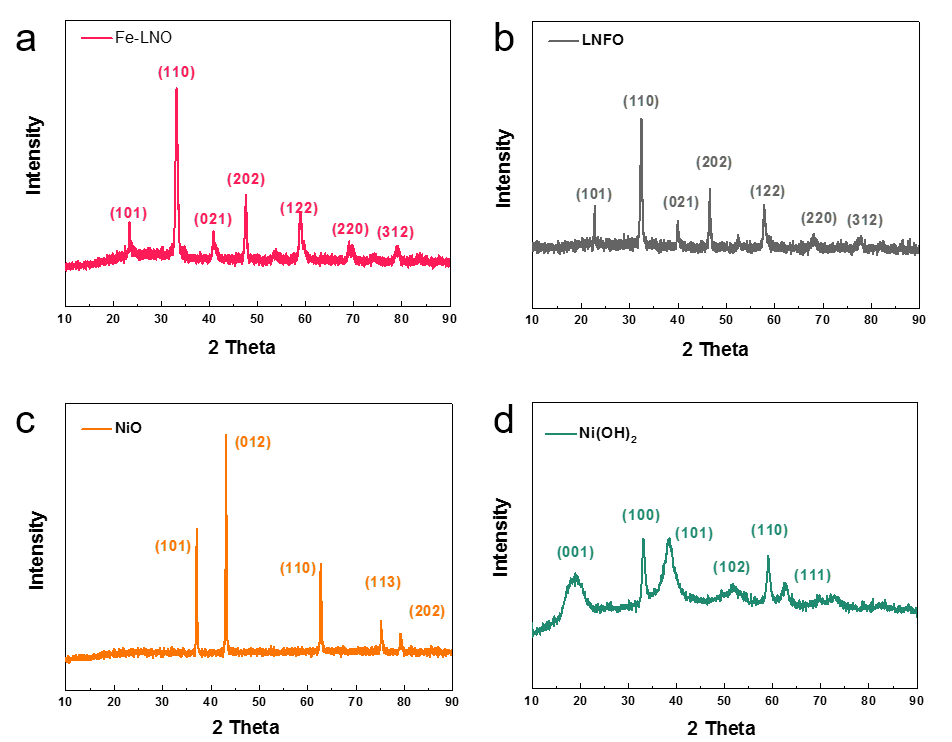


**Figure S9.** XRD patterns of Fe-LNO (a), LNFO (b), NiO (c), and Ni(OH)_2_ (d).


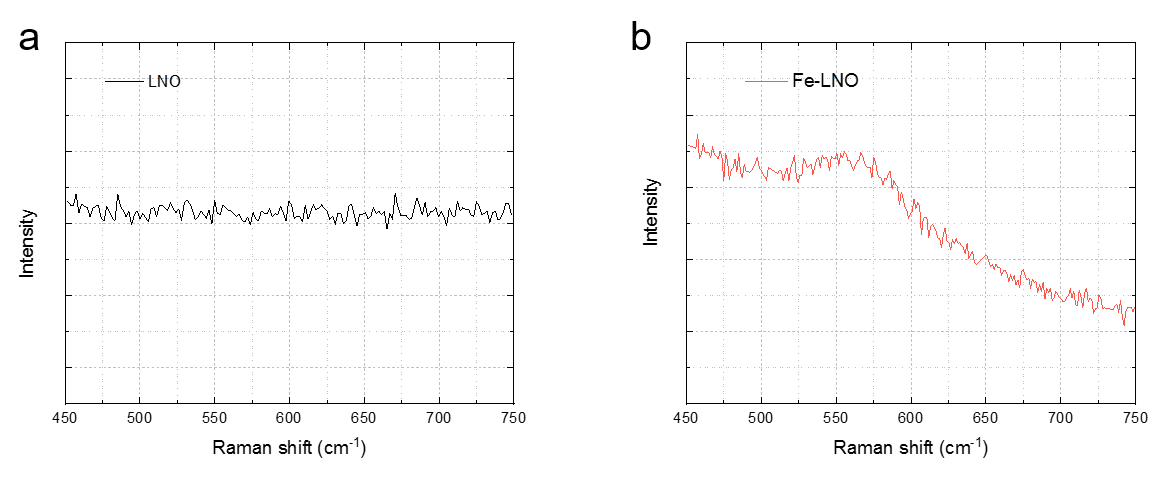


**Figure S10.** Raman spectra of LaNiO_3_ (a) and Fe-LNO (b). The peak at around 560 cm^-1^ is closest in shift to α-FeOOH.


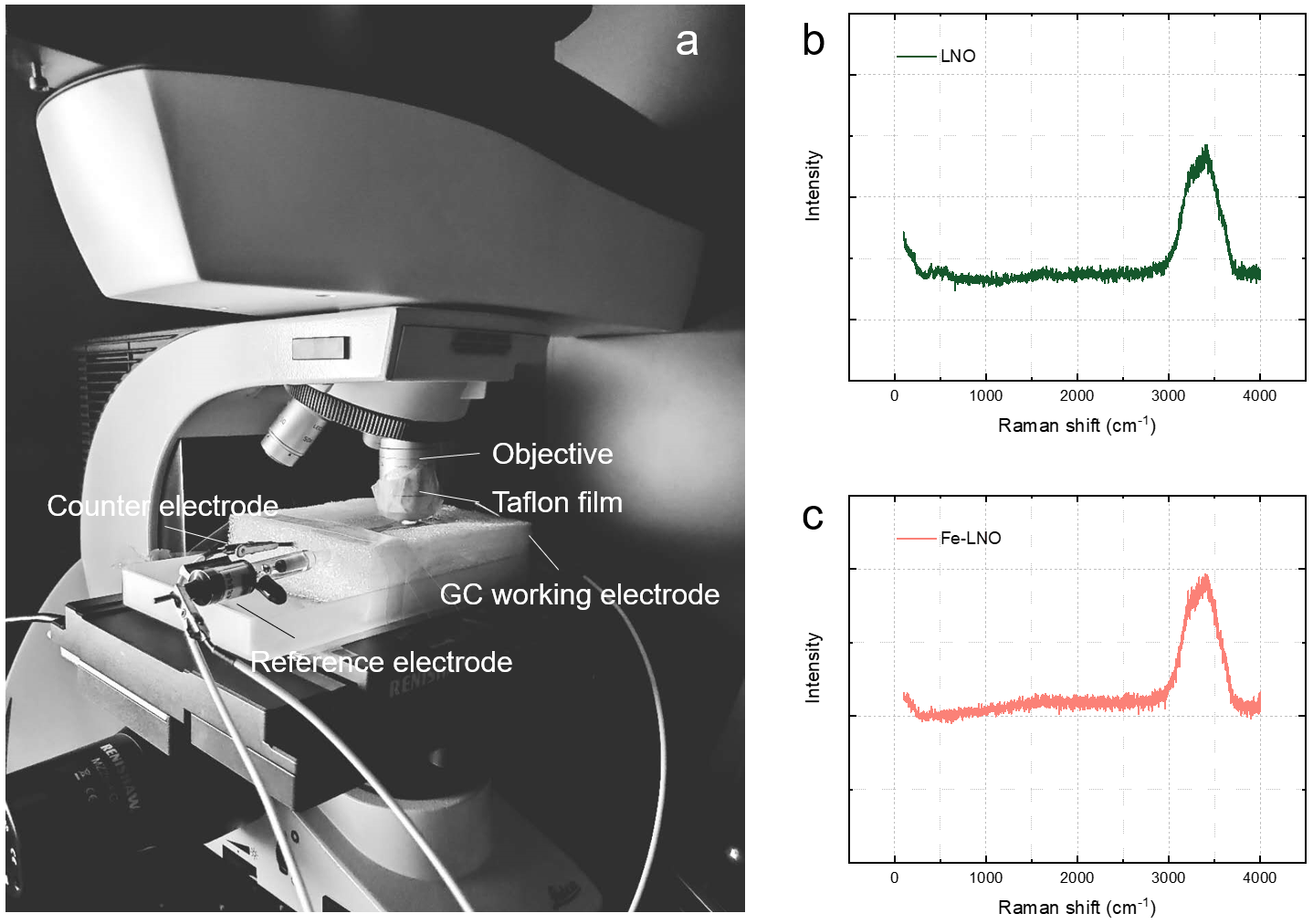


**Figure S11.** (a) In situ Raman spectroscopic device photograph. The in situ Raman spectra of (b) LNO and (c) Fe-LNO at 1.73 V vs RHE.


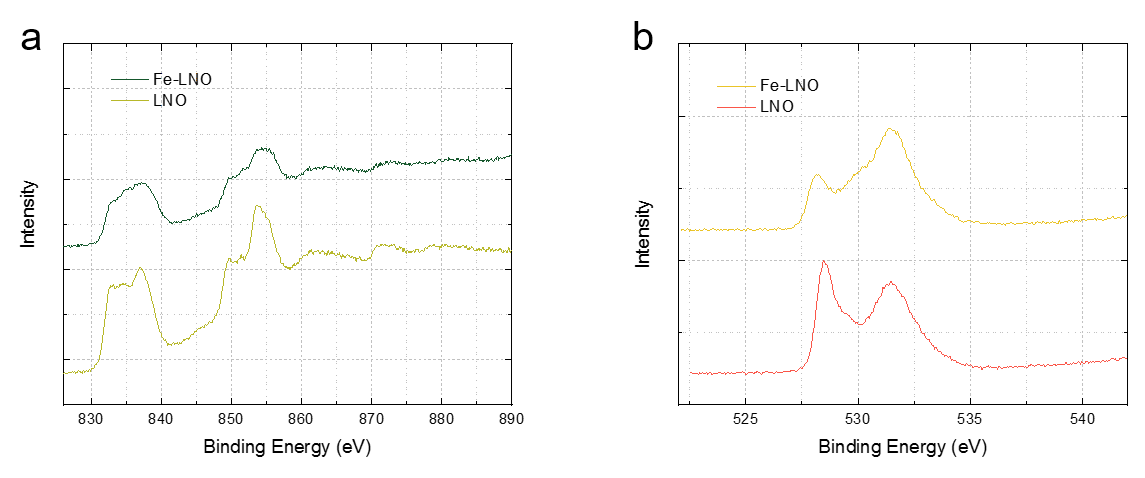


**Figure S12.** High-resolution XPS spectra of (a) Ni 2p, La 3d, and (b) O 1s of LNO and Fe-LNO.


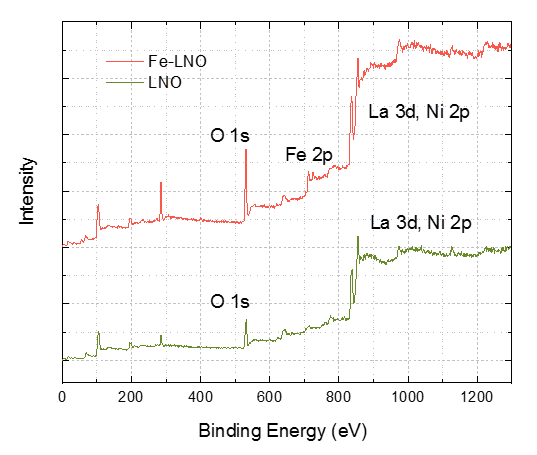


**Figure S13.** The XPS survey spectra of the as‐synthesized LNO and Fe-LNO.


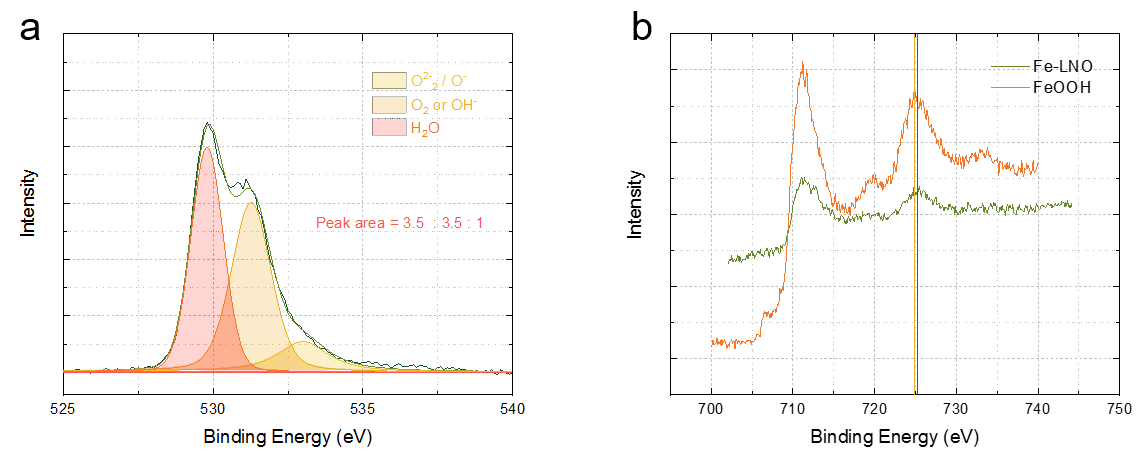


**Figure S14.** (a) High-resolution O 1s XPS spectra of FeOOH. The ratios of peak areas of H_2_O: OH^-^:O_2_^2-^ is about 3.5 : 3.5 : 1, which is higher than LaNiO_3_ but lower than Fe-LNO, proved that hydroxyl adsorption is not only came from FeOOH; (b) High-resolution Fe 2p XPS spectra of FeOOH and Fe-LNO.


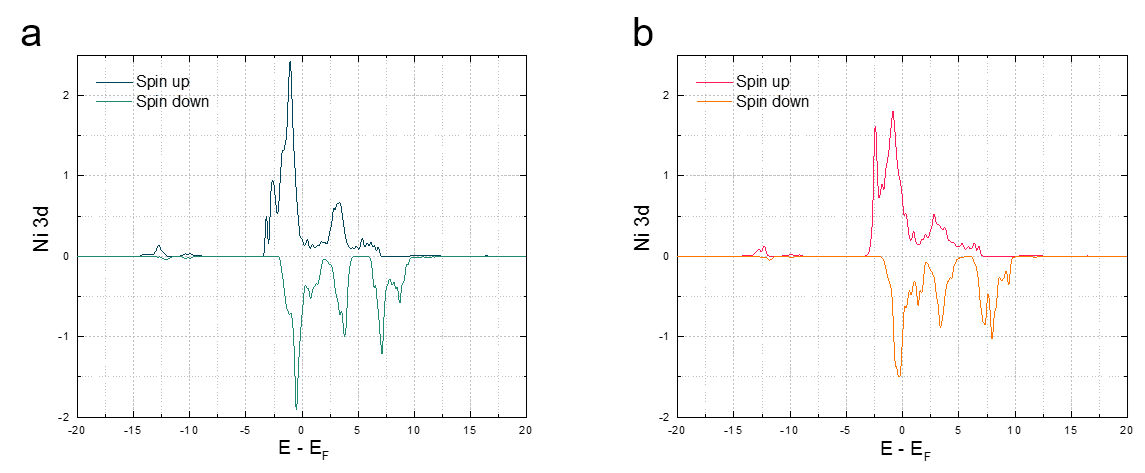


**Figure S15.** Projected density of states of Ni (3*d*) orbitals in bulk NiO_5_ (a) and surface NiO_5_ (b). All calculation results were normalized with Fermi level.


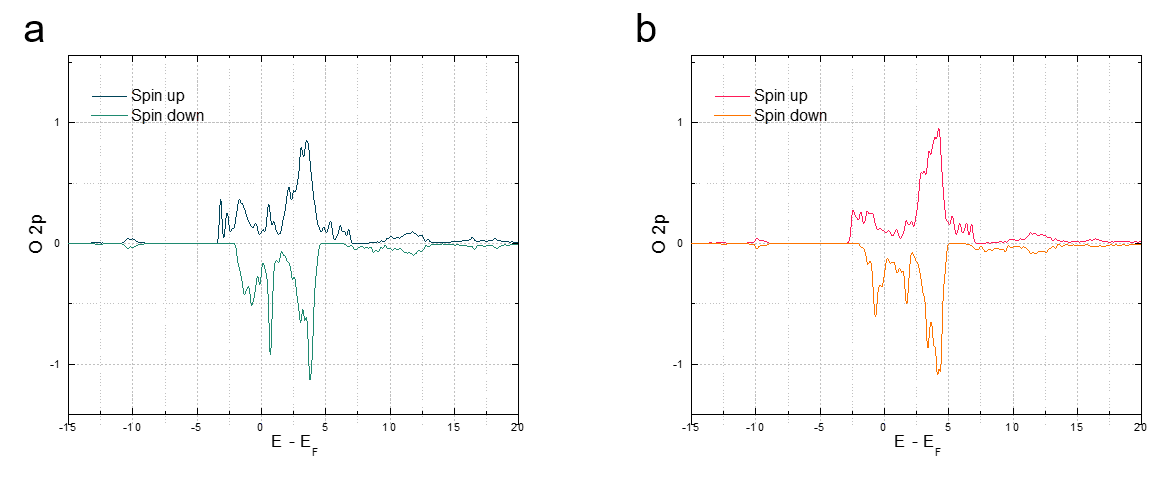


**Figure S16.** Projected density of states of O (2*p*) orbitals in bulk NiO_5_ (a) and surface NiO_5_ (b). All calculation results were normalized with Fermi level.


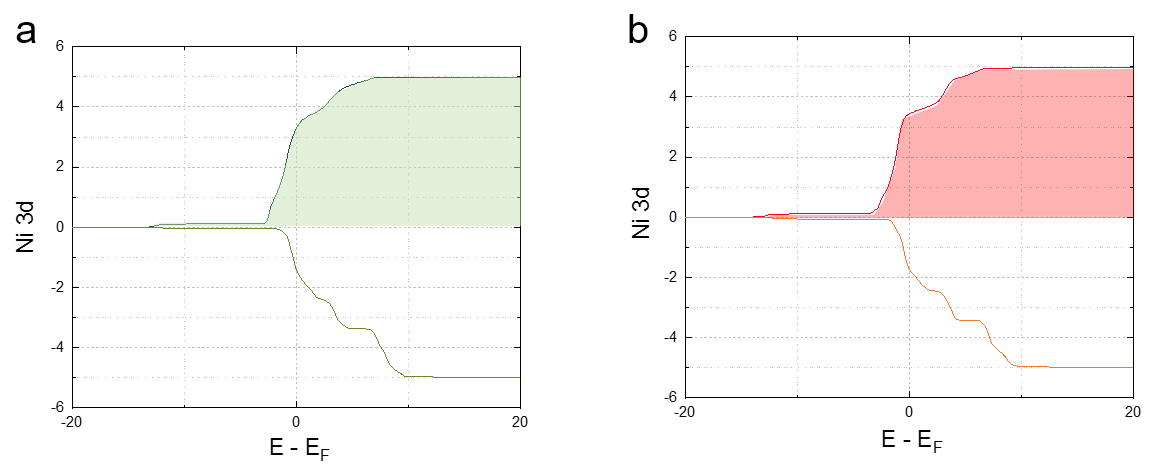


**Figure S17.** Integral curve of Ni 3d density of states in bulk NiO_5_ (a) and surface NiO_5_ (b).


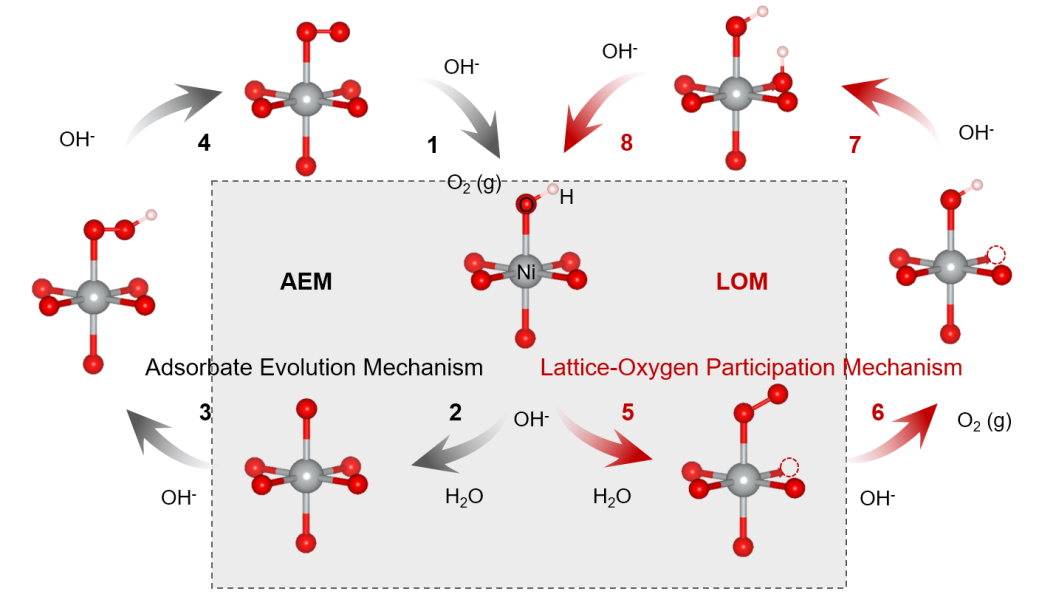


**Figure S18.** The proposed two OER mechanisms of LNO with the same starting point (Ni-*OH). The dashed box represents the most important step in the adsorbate evolution mechanism and lattice-oxygen participation mechanism.


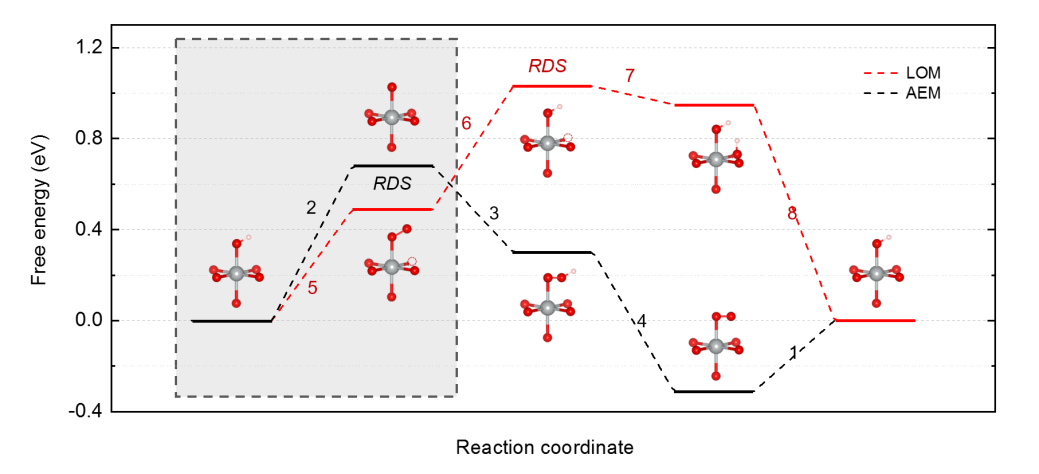


**Figure S19.** The Free energies of LNO with the same starting point (the dashed box represents the most important step in adsorbate evolution mechanism and lattice-oxygen participation mechanism).


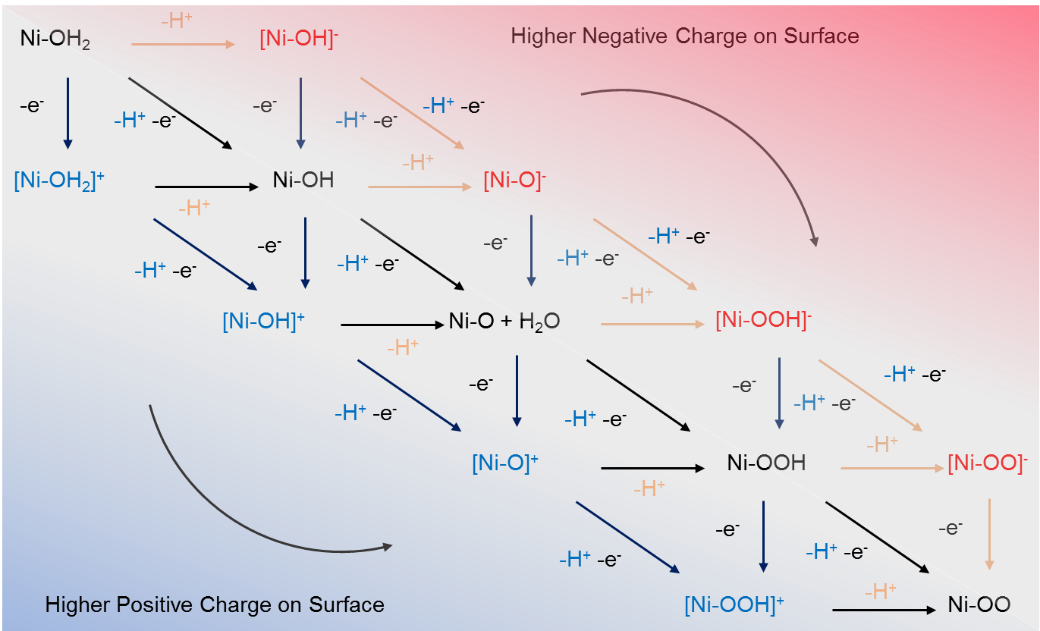


**Figure S20.** Four concerted proton-coupled electron transfer mechanism for OER occurring at Ni site (Ni) (in red) and possible sequential proton-electron transfers along the path, constructed with the assumption that the charge of the reaction intermediates does not exceed one electron.


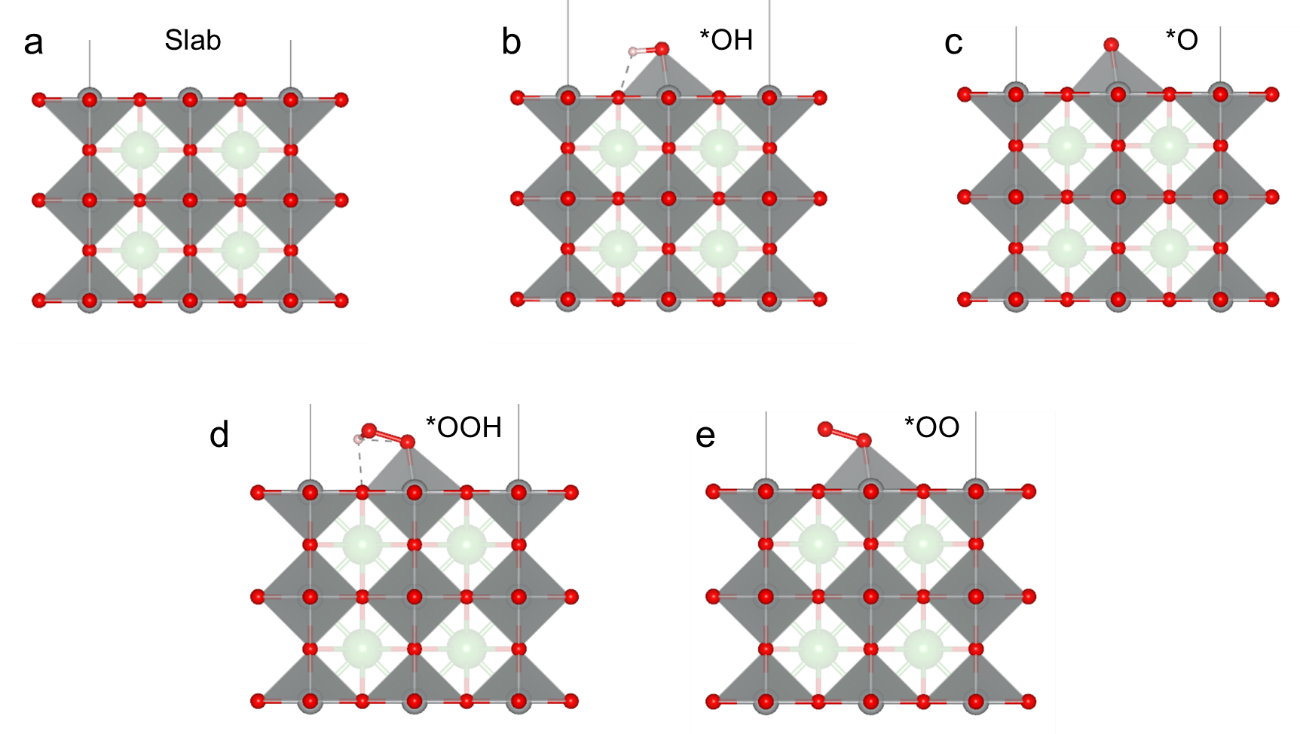


**Figure S21.** Oxygen evolution reaction pathway of LNO, slab model (a), M-OH (b), M-O (c), M-OOH (d), M-OO (e). All the structures are calculated in a same parameter.


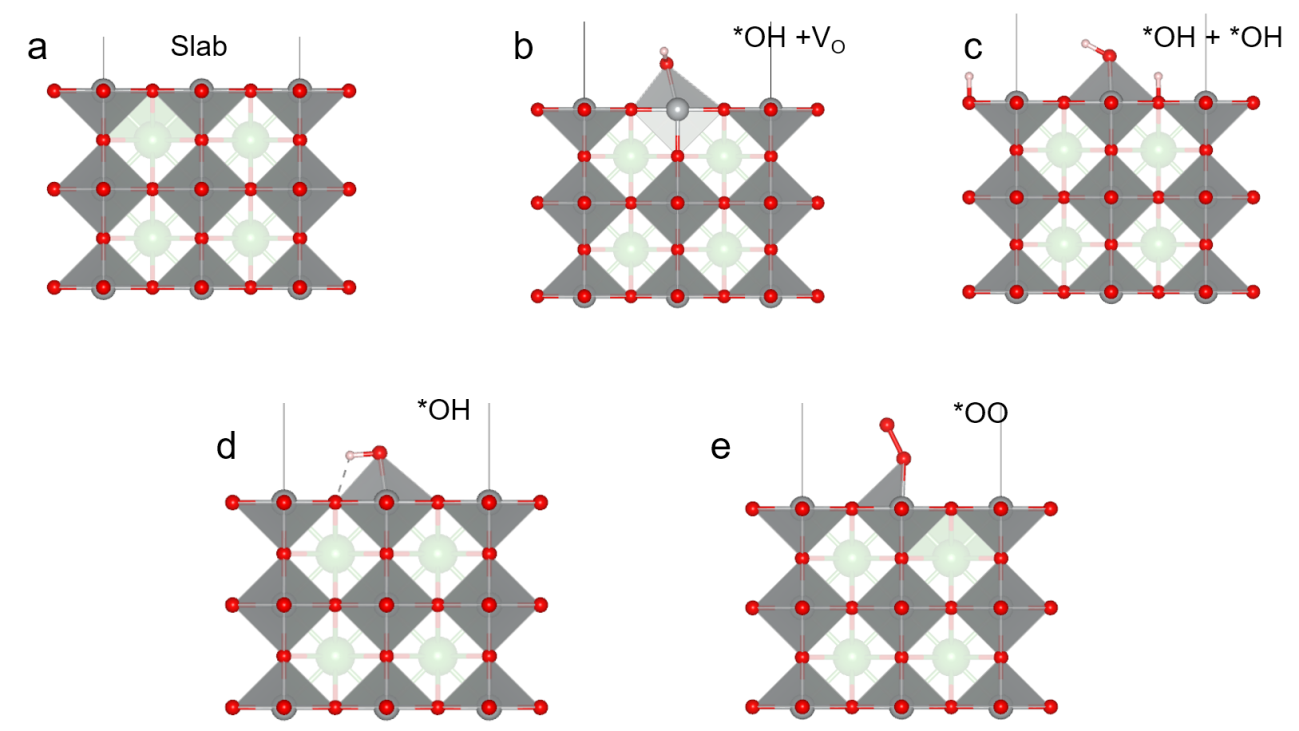


**Figure S22.** Lattice oxygen participated oxygen evolution reaction pathway of LNO, slab model with lattice oxygen vacancy (a), M(O_v_)-OH +V_O_ (b), M(O_v_)-*OH-*OH (c), M(O_v_)-*OH (d), M(O_v_)-*OO (e). All the structures are calculated in a same parameter.


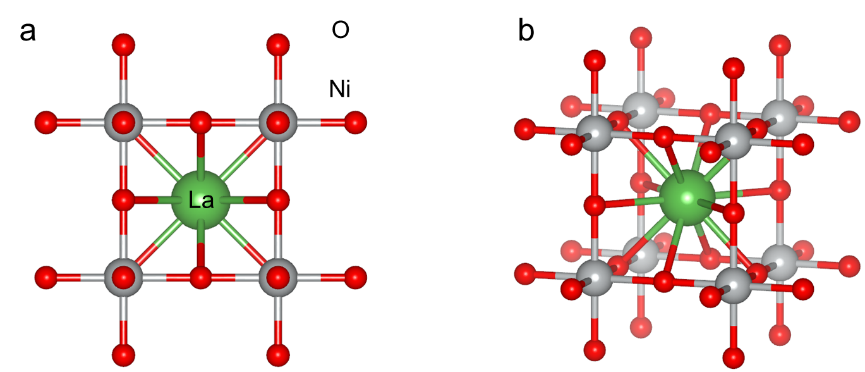


**Figure S23.** Bulk models of LNO with top view (a) and side view (b). (The green, red, and gray balls represent La, O and Ni atoms, respectively. The lattice constant of unit cell is a = b = c = 3.857 Å).


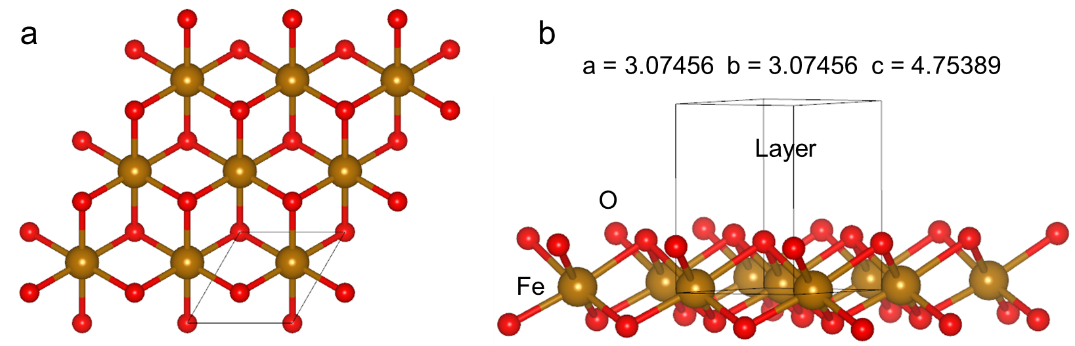


**Figure S24.** Bulk models of FeOOH with top view (a) and side view (b). (The brown and red balls represent Fe and O atoms, respectively.)


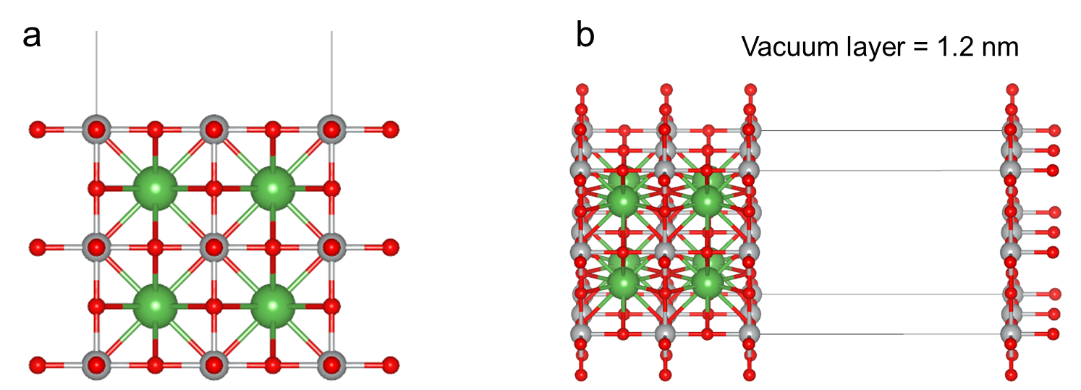


**Figure S25.** Slab models of LNO with side view (a) and perspective view (b). The vacuum layer was 12 Å. The top layer of NiO_5_ is relax while other inner layers is fixed.


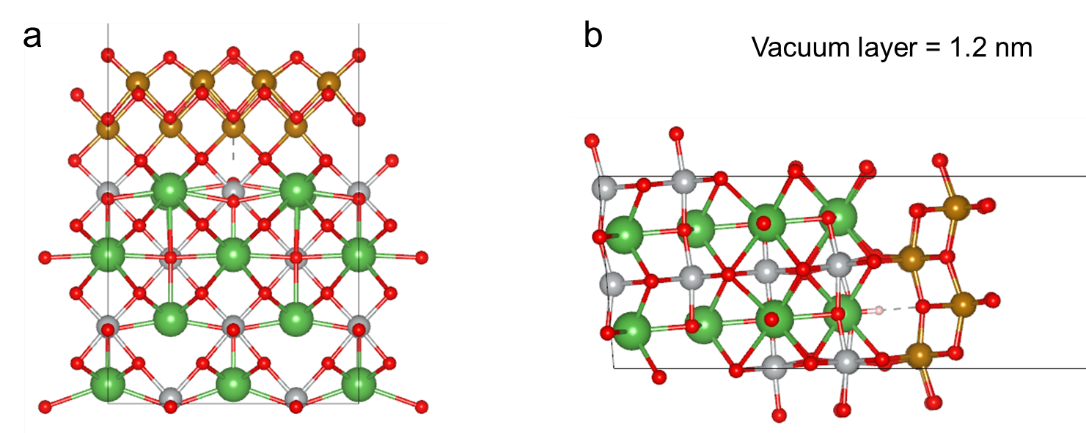


**Figure S26.** Slab models of Fe-LNO with side view (a) and left view (b). The vacuum layer was 12 Å. The whole FeOOH layer and the top layer of NiO_5_ is relax while other inner layers is fixed. The calculation of *OH adsorption energy is simulated by bulk catalysis.


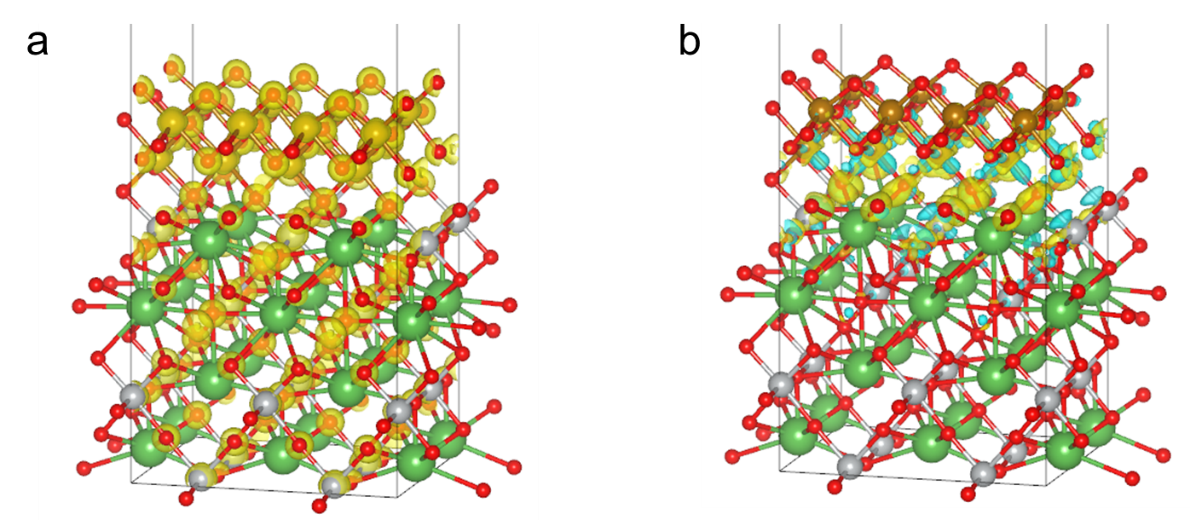


**Figure S27.** DFT calculated electronic structures for Figure 1. (a) The charge distribution of Fe-LNO; (b) the charge density difference on Fe-LNO: ρ= ρ[Fe-LNO] + ρ(FeOOH) - ρ[LaNiO_3_]; Yellow and cyan iso-surface represents electron accumulation and electron depletion, respectively.


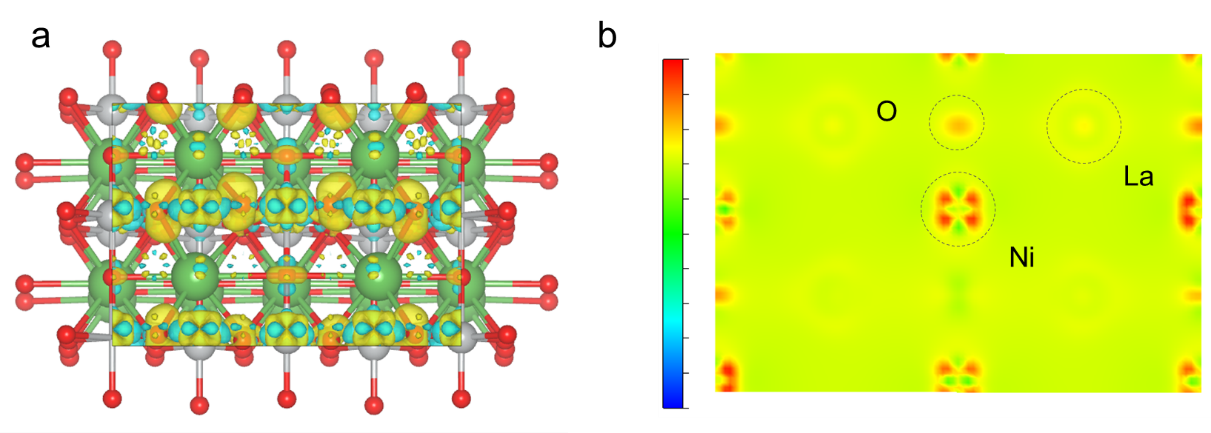


**Figure S28.** (a) Top view of the charge density difference on Fe-LNO. (b) Iso-surface 2D view of the surface NiO_5_.


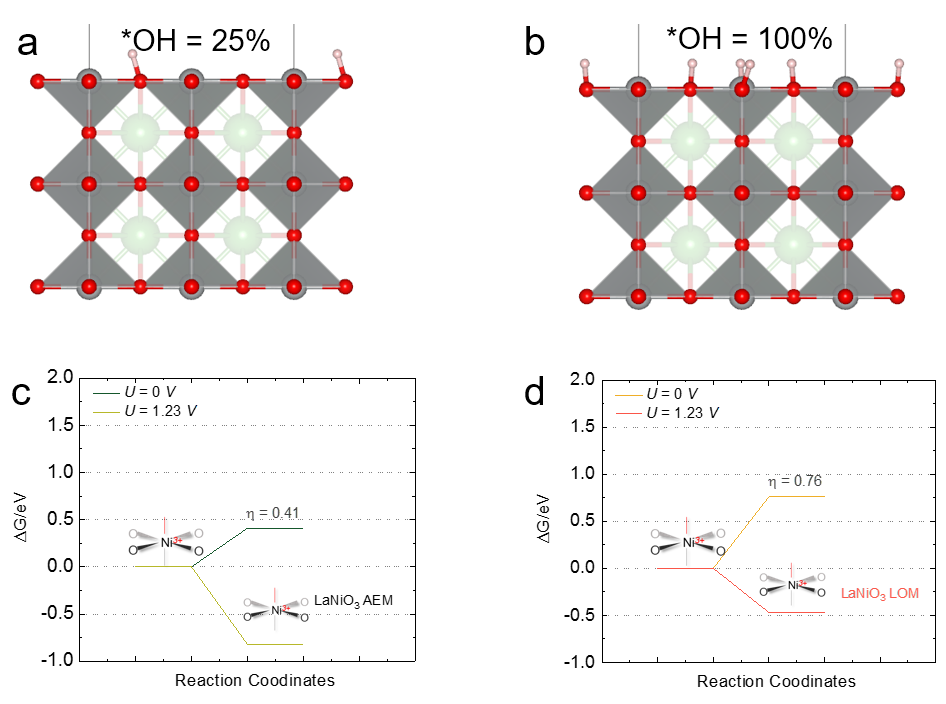


**Figure S29.** The free energy of *OH adsorption in LNO with different *OH coverage, 25% (a) ~ 100% (b). The related change of free energy of reaction (c, d). The coverage of 100% is using single unit cell to construct, while the 25% is using super cell with 2*2 in xy axis.


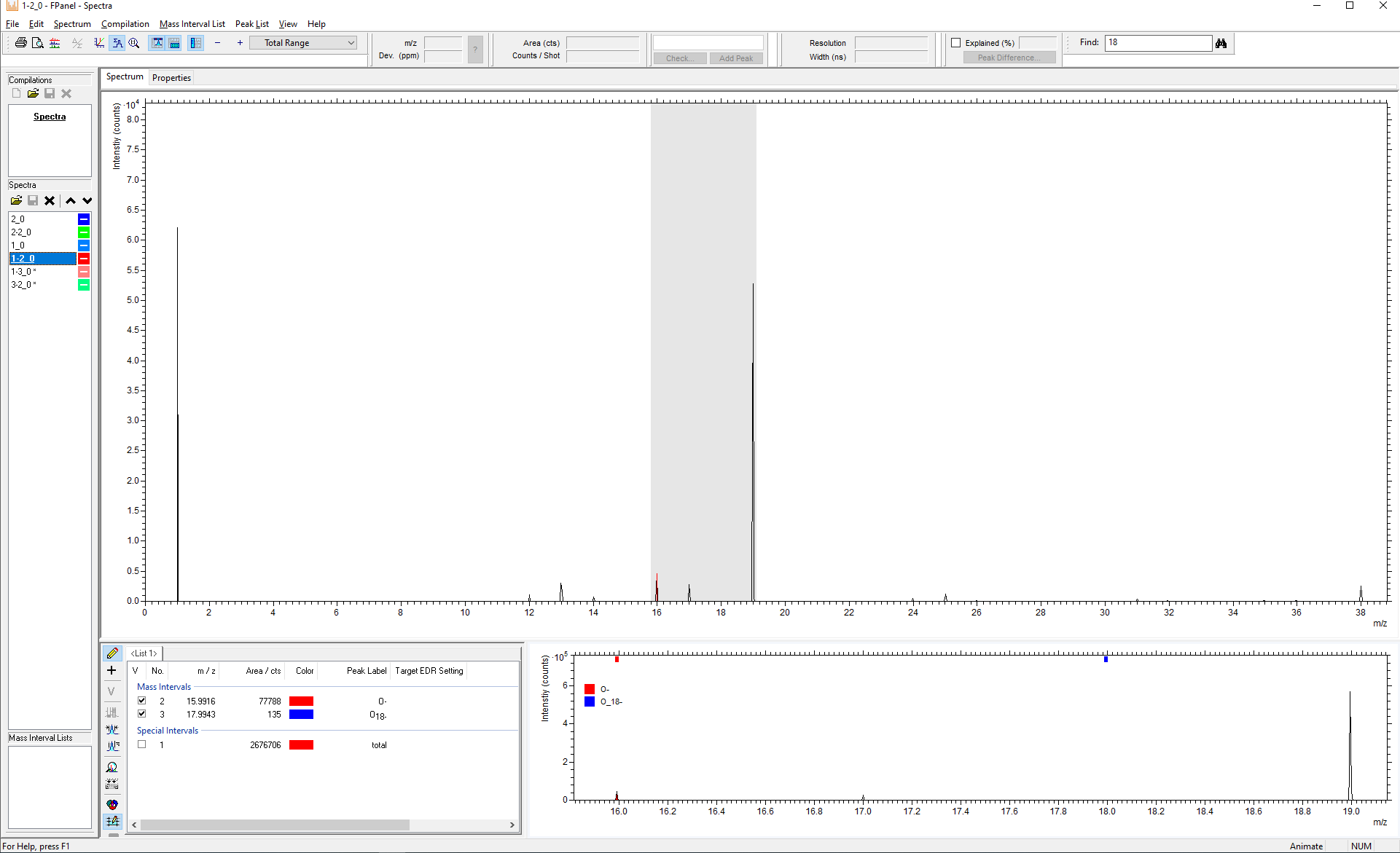


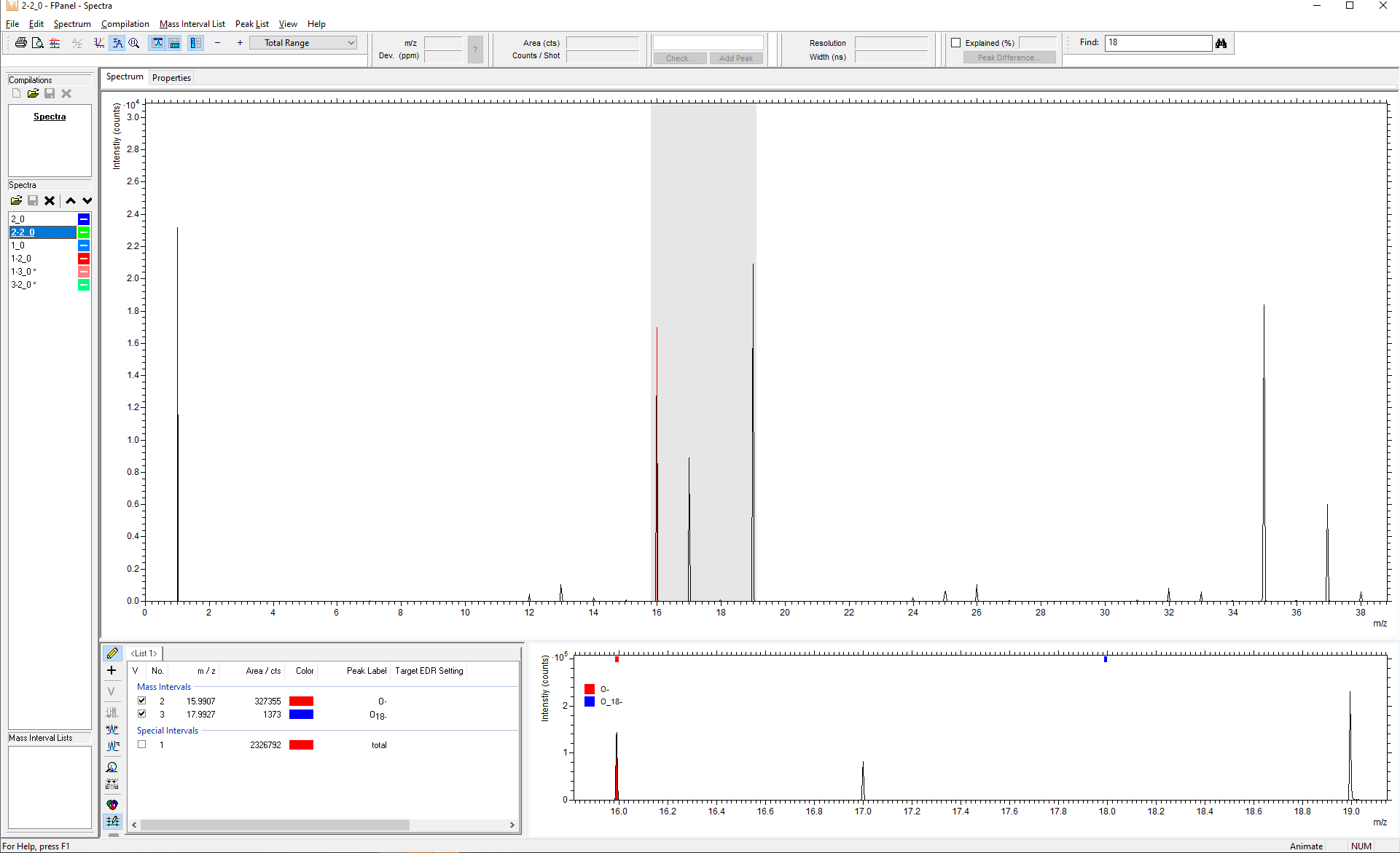


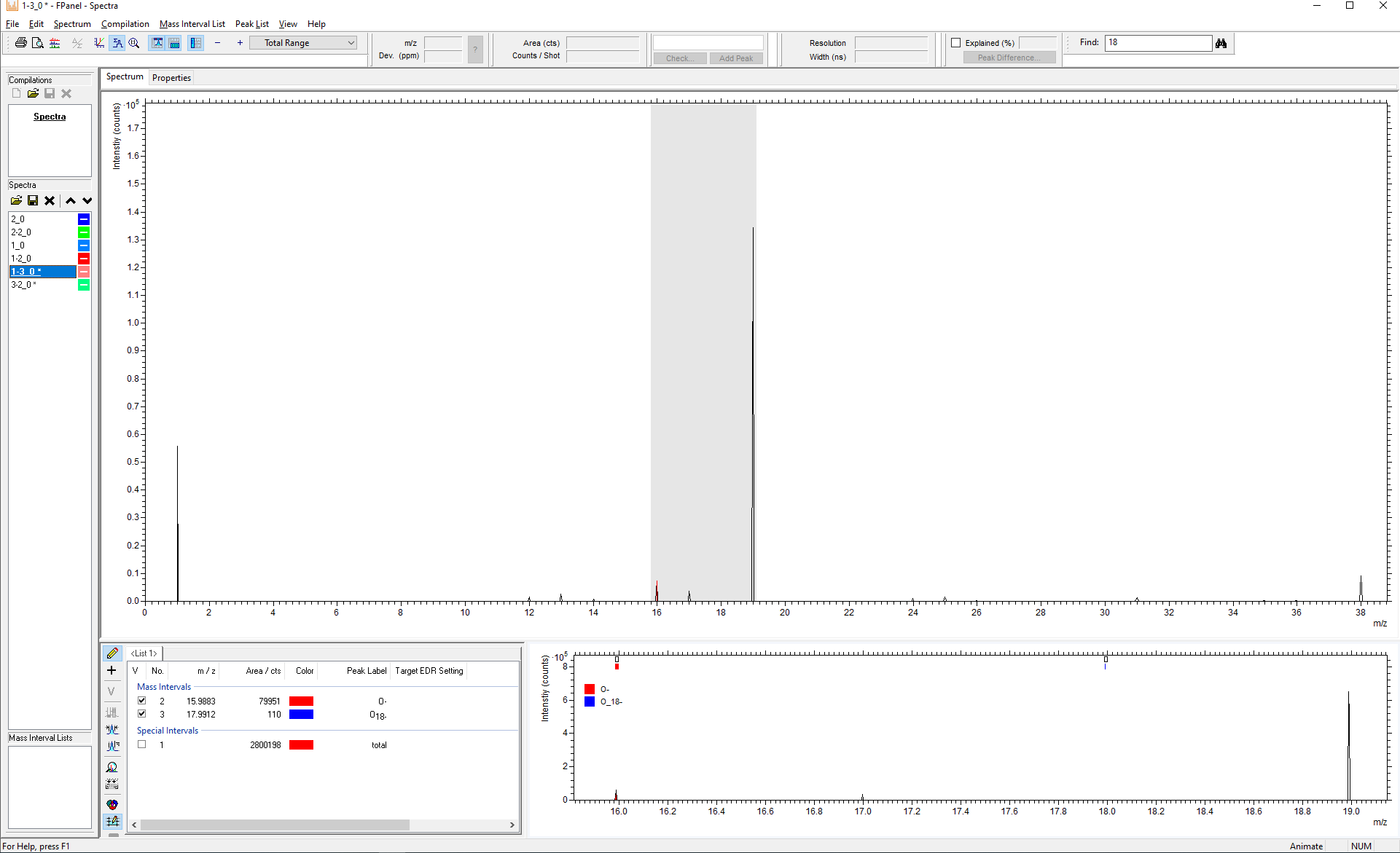


**Figure S30.** The images of SIMS tests of LNO (top), Fe-LNO (middle), and FeOOH (bottom).


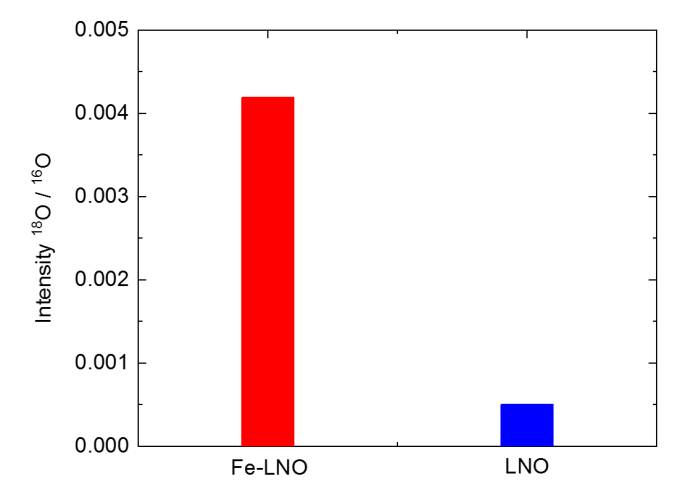


**Figure S31.** The area ratio of Fe-LNO and LNO supported on FeOOH.


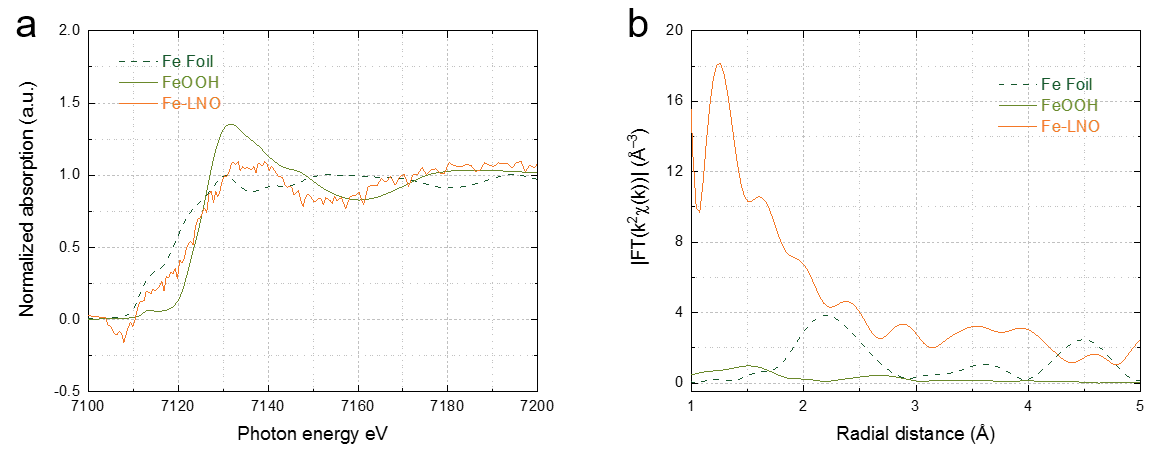


**Figure S32.** (a) Normalized Fe K-edge XANES spectra and (b) EXAFS *k*^2^χ(*k*) Fourier transform (FT) spectra of FeOOH and Fe-LNO with Fe foil as a reference.


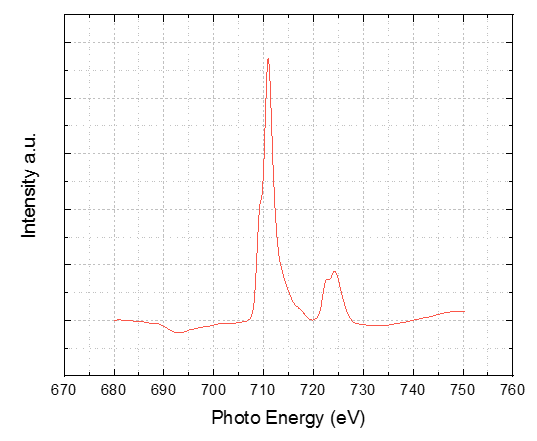


**Figure S33.** The soft-XAFS spectrum of Fe-LNO.


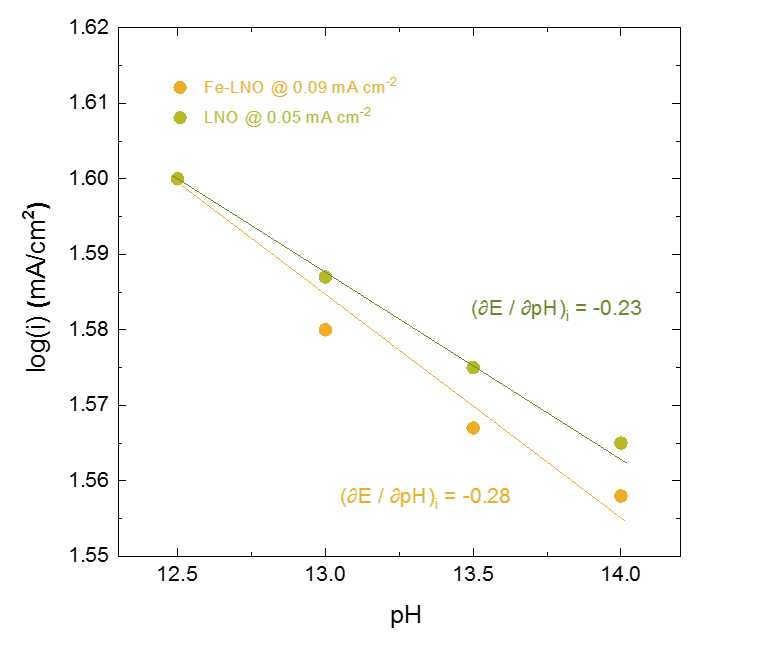


**Figure S34.** Specific OER catalytic activity (current normalized by geometry area) at 1.60 V (vs RHE) as a function of pH.


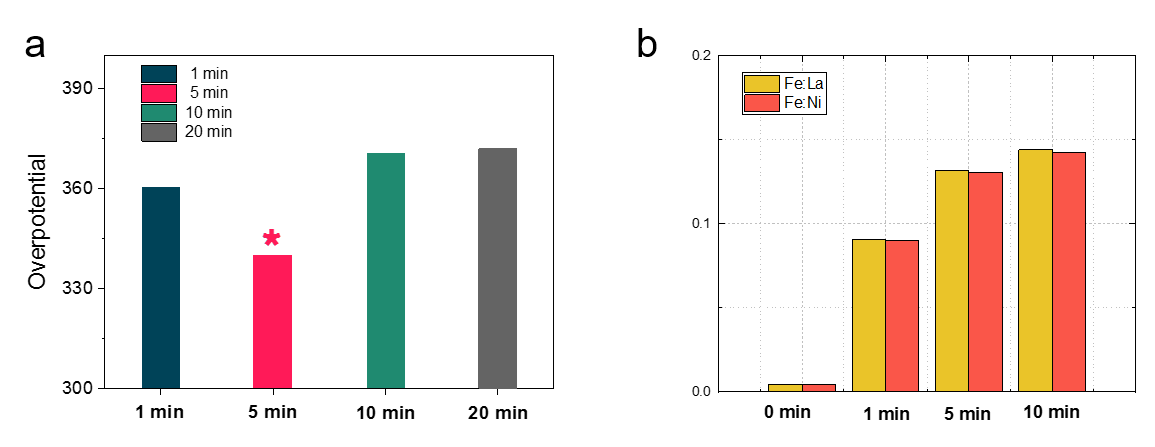


**Figure S35.** (a) The effect of different deposition time of Fe on catalytic performance and (b) the ratio of Fe to Ni at different deposition time. The ratio is obtained by ICP-AES, and it can be found that the ratio of Fe to Ni is the best at 0.12.


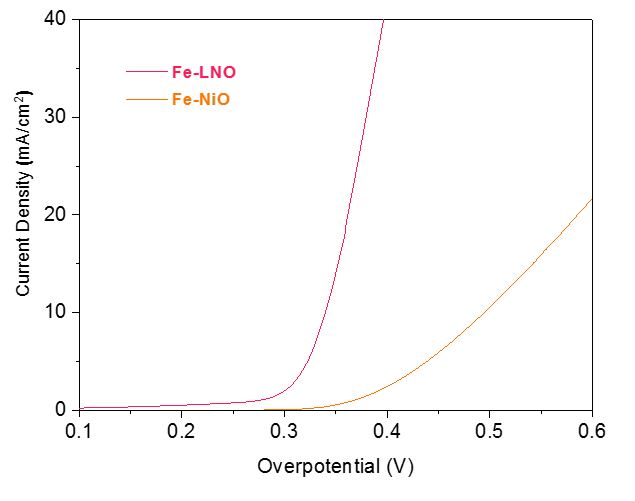


**Figure S36.** The OER activities of FeOOH-NiO and Fe-LNO normalized to geometry area. NiO powder was synthesized using a Pechini method, followed by crystallization and annealing, as the same procedure of LaNiO_3_.


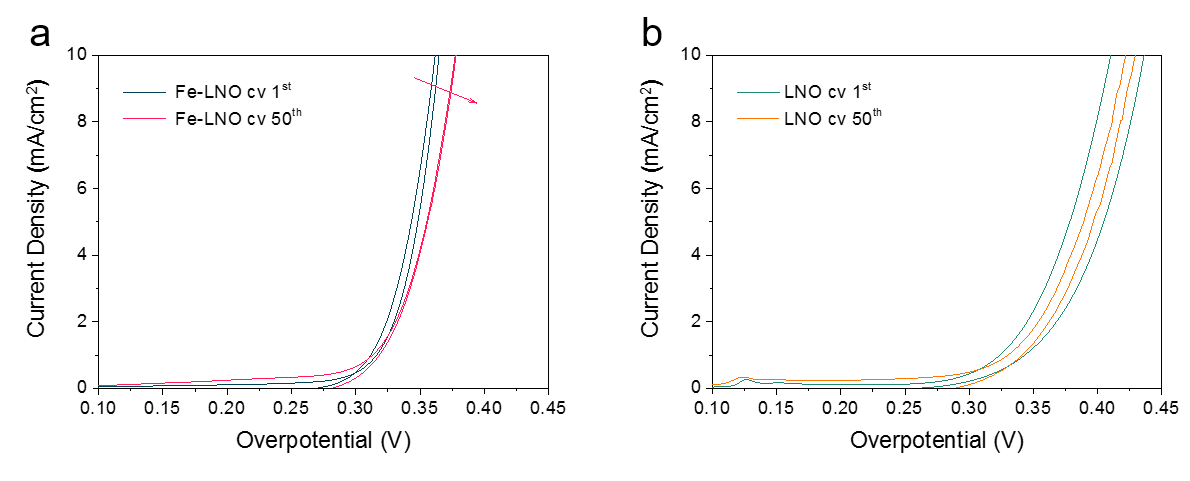


**Figure S37.** Cyclic voltammetry stability tests of Fe-LNO (a) and LNO (b). A total of 50 cycles (1.27 V ~ 1.62 V vs RHE) at a scan rate of 10 mV s^-1^ in 1.0 M KOH.


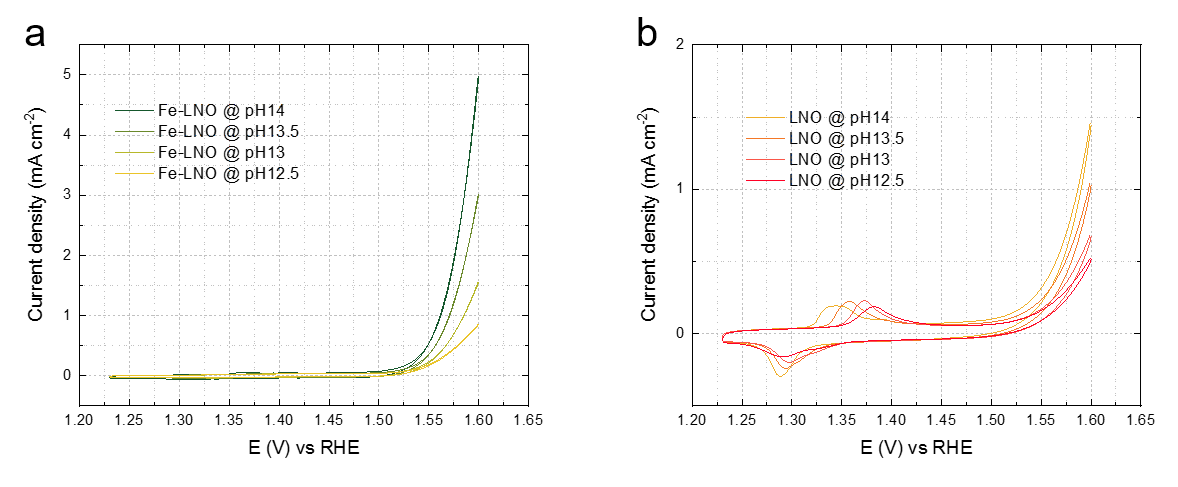


**Figure S38.** CV measurements of KOH solution with different pH values recorded on (a) Fe-LNO and (b) LNO at 10 mV s^–1^. All the KOH was used in metal basis 99.999%. The catalyst inks were mixed by 3 mg catalysts powder with 1 ml NaOH neutralized 0.05 wt% Nafion solution and bath sonicated for at least half an hour. Three microliters of catalyst ink were drop cast onto 3 mm glassy carbon electrodes (GCE).


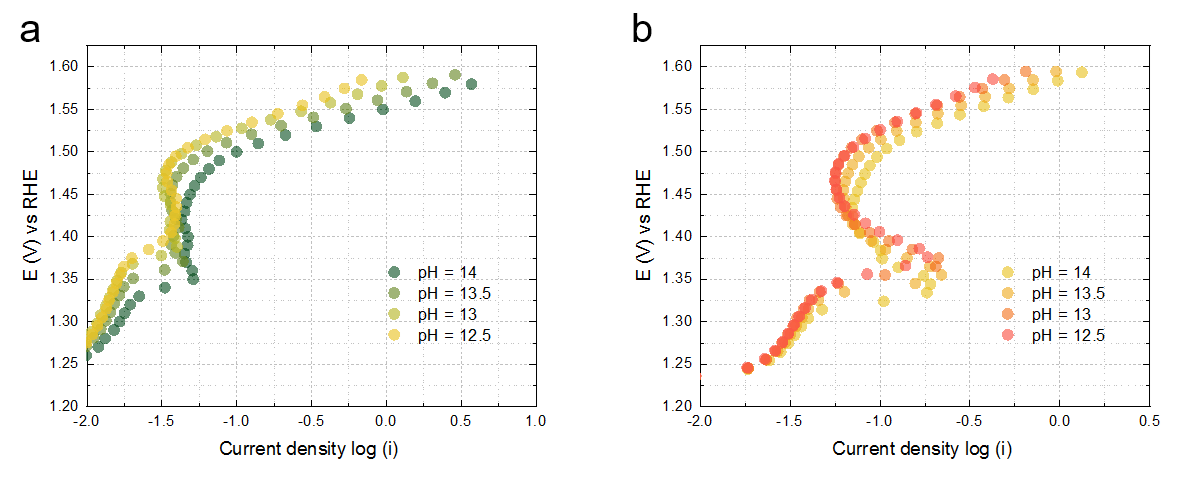


**Figure S39.** CV measurements from 0.03 M KOH (pH 12.5) to 1 M KOH (pH=14) recorded at 10 mV s^–1^: (a) Fe-LNO; (b) LNO (All the KOH was used in metal basis 99.999%).


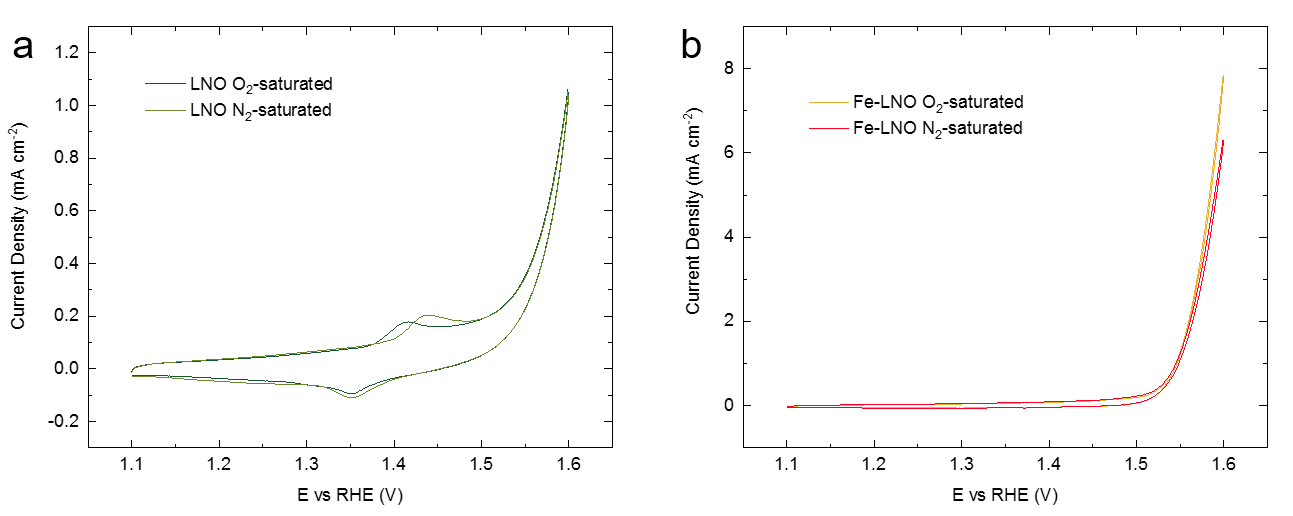


**Figure S40.** The OER catalytic activities of catalysts in 1.0 M KOH solution saturated with O_2_ and N_2_: (a) LNO, (b) Fe-LNO.


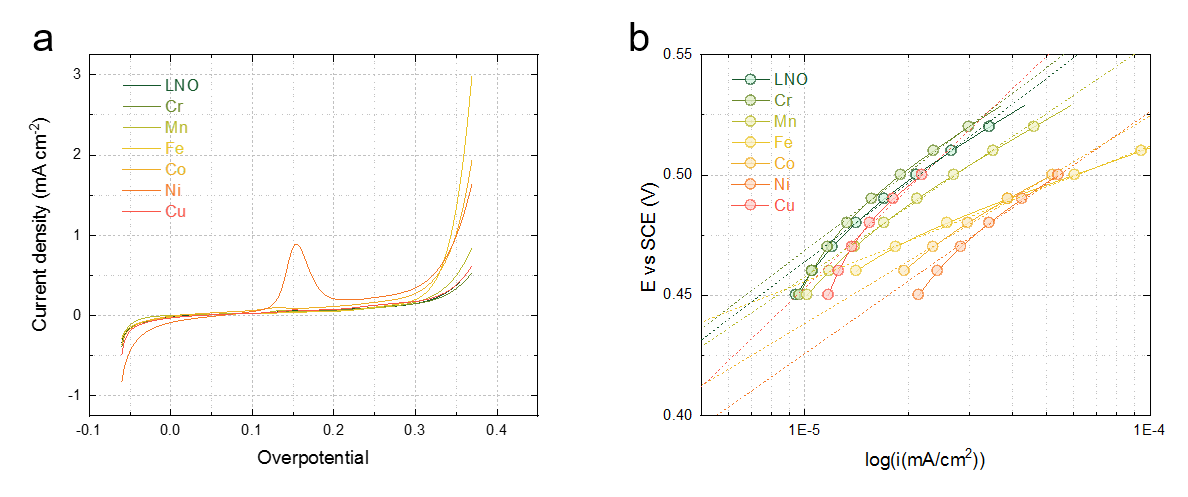


**Figure S41.** (a) The OER catalaytic activities of various samples (LaNiO_3_, CrO_x_H_y_-LaNiO_3_, MnO_x_H_y_-LaNiO_3_, FeO_x_H_y_-LaNiO_3_, CoO_x_H_y_-LaNiO_3_, NiO_x_H_y_-LaNiO_3_, and CuO_x_H_y_-LaNiO_3_) normalized to the geometry area at a scan rate of 5 mV s^-1^ in 1.0 M KOH. (b) The related Tafel curves of various samples.

Here MO_x_H_y_-LaNiO_3_ (M=Cr, Mn, Fe, Co, Ni, or Cu) was obtained by a simple deposition way as shown follows: 0.246 g of LaNiO_3_ sample was immersed into a 100 mL aqueous solution containing 2.78 g of metal sulfate or nitrate and then was stirred under room temperature for 5 min. The pH of above solution must be hold around 4 to prevent LaNiO_3_ from being etched. Then the above solution was centrifuged for three times, and the obtained solid powders were further oxidized at 70 ^o^C in air to synthesize MO_x_H_y_-LaNiO_3_. The catalyst inks were obtained by mixing 3 mg catalyst powders with 1 ml NaOH solution (1.0 M) and then were neutralized with 0.05 wt% Nafion solution. The catalyst inks must be bath sonicated for at least half an hour before use. Three microliters of catalyst inks were dropped onto 3 mm glassy carbon electrodes (GCE) to prepare the working electrode. The above results show that MnO_x_H_y_-LaNiO_3_, FeO_x_H_y_-LaNiO_3_, CoO_x_H_y_-LaNiO_3_, and NiO_x_H _y_-LaNiO_3_ can optimize the OER activity and kinetics of pristine LaNiO_3_, which prove that the MO_x_H_y_ can promote the adsorption of hydroxyl to boost LOER of perovskites.


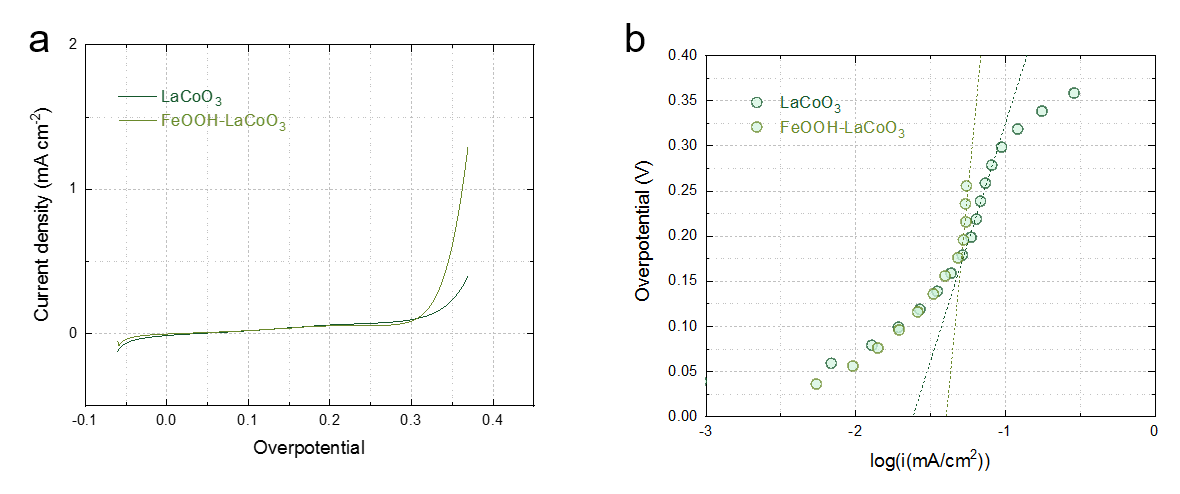


**Figure S42.** (a) The OER catalytic activities of samples LaCoO_3_ and FeOOH-LaCoO_3_ normalized to geometry area at a scan rate of 5 mV s^-1^ in 1 M KOH; (b) The related Tafel curve of samples.


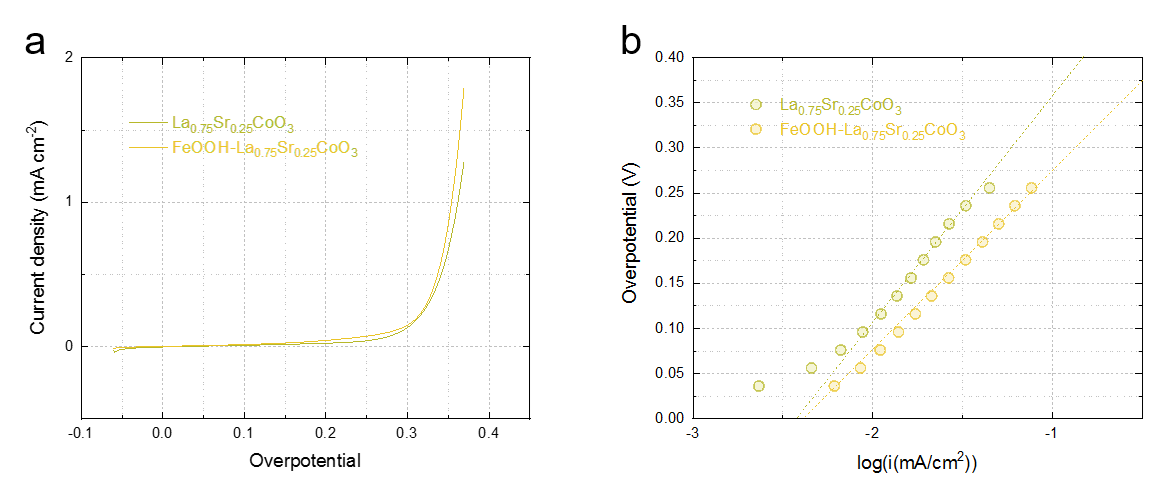


**Figure S43.** (a) The OER catalytic activities of samples La_0.75_Sr_0.25_CoO_3_ and FeOOH-La_0.75_Sr_0.25_CoO_3_ normalized to geometry area at a scan rate of 5 mV s^-1^ in 1.0 M KOH; (b) The related Tafel curve of samples.


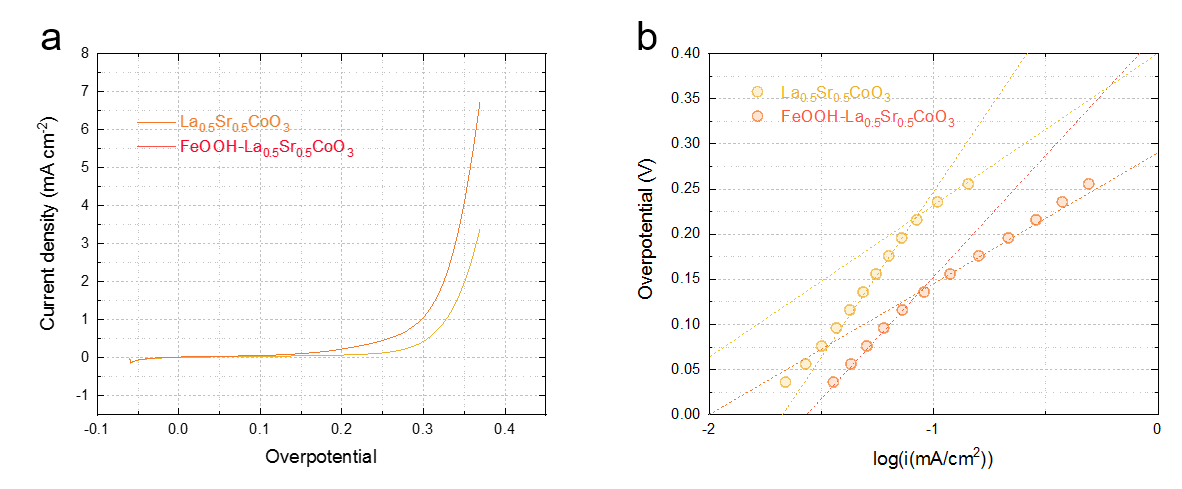


**Figure S44.** (a) The OER catalytic activities of samples La_0.5_Sr_0.5_CoO_3_ and FeOOH-La_0.5_Sr_0.5_CoO_3_ normalized to geometry area at a scan rate of 5 mV s^-1^ in 1 M KOH; (b) The related Tafel curve of samples.


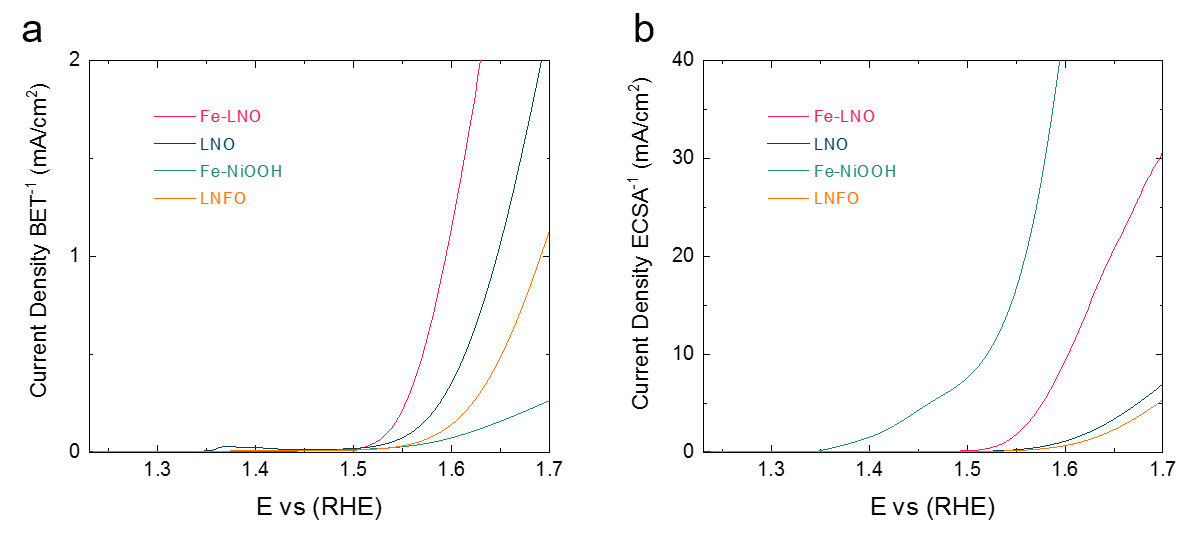


**Figure S45.** The OER activities of samples Fe-LNO, LNO, Fe-NiOOH, LNFO normalized to BET surface area (a) and ECSA (b). The conversion method is by dividing the geometric area directly by the corresponding ECSA and BET values.


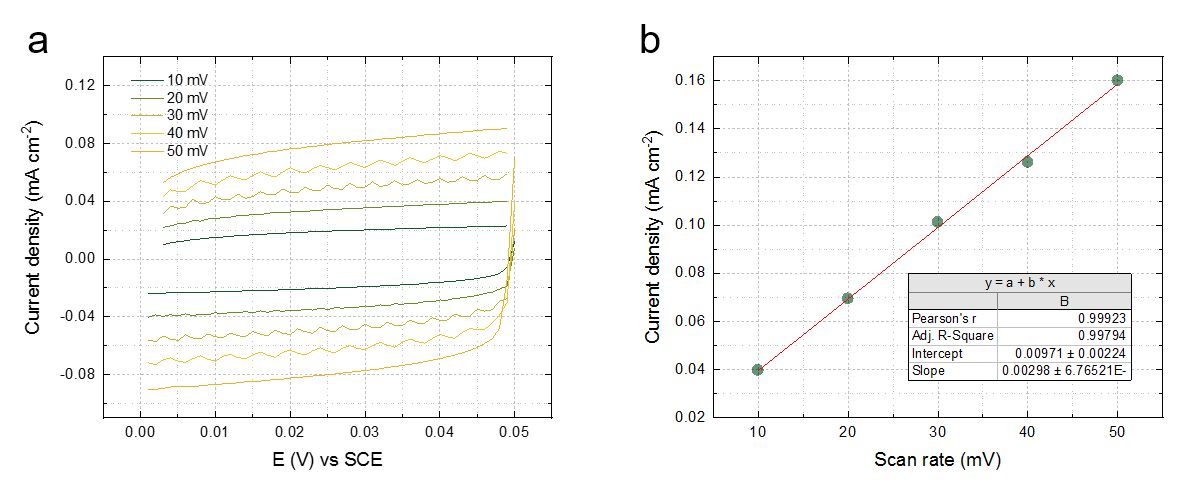


**Figure S46.** Double-layer capacitance measurements for determining electrochemically active surface area (ECSA) of LNO from cyclic voltammetry in 1.0 M KOH solution. (a) Typical cyclic voltammograms in the non-Faradaic region around 0.05 V relative to SCE at different scan rates (0.01, 0.02, 0.03, 0.04, 0.05 V s^-1^ ); (b) the average of the cathodic and anodic charging currents absolute values measured at 0.025 V (vs. SCE) plotted as a function of scan rate.


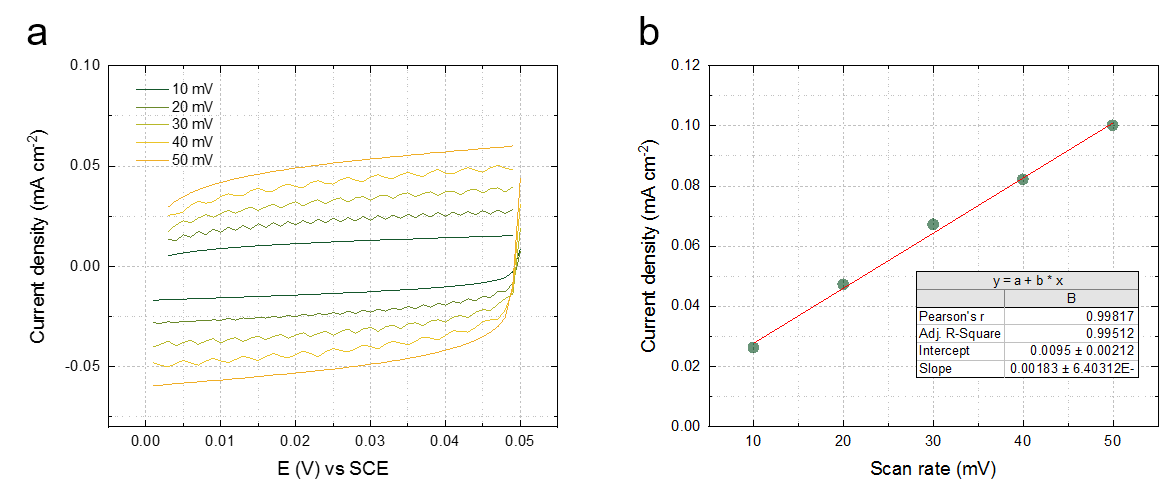


**Figure S47.** Double-layer capacitance measurements for determining electrochemically active surface area (ECSA) of Fe-LNO from cyclic voltammetry in 1.0 M KOH solution. (a) Typical cyclic voltammograms in the non-Faradaic region around 0.05 V relative to SCE at different scan rates (0.01, 0.02, 0.03, 0.04, 0.05 V s^-1^ ); (b) the average of the cathodic and anodic charging currents absolute values measured at 0.025 V (vs. SCE) plotted as a function of scan rate.


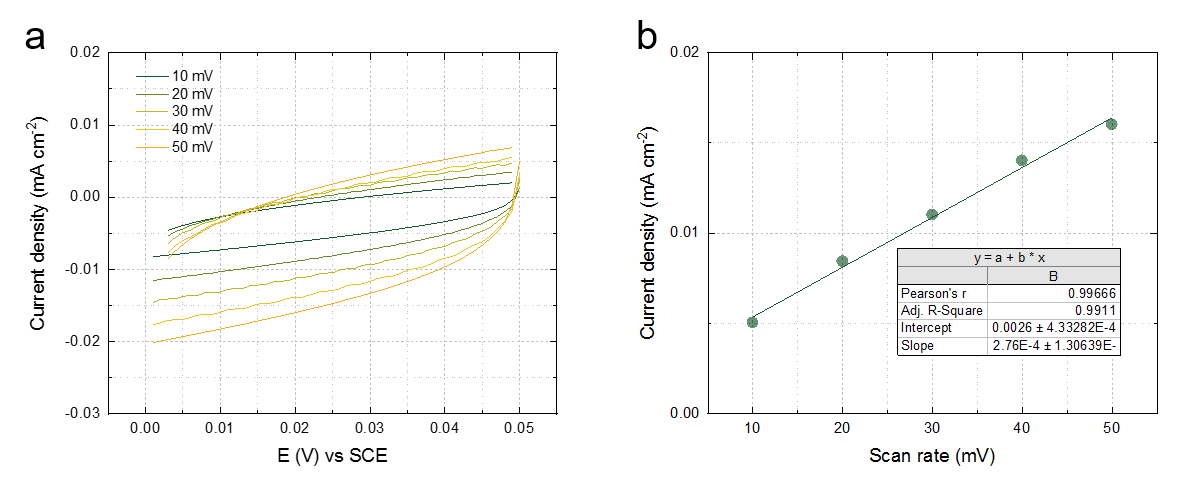


**Figure S48.** Double-layer capacitance measurements for determining electrochemically active surface area (ECSA) of Fe-NiOOH from cyclic voltammetry in 1.0 M KOH solution. (a) Typical cyclic voltammograms in the non-Faradaic region around 0.05 V relative to SCE at different scan rates (0.01, 0.02, 0.03, 0.04, 0.05 V s^-1^ ); (b) the average of the cathodic and anodic charging currents absolute values measured at 0.025 V (vs. SCE) plotted as a function of scan rate.


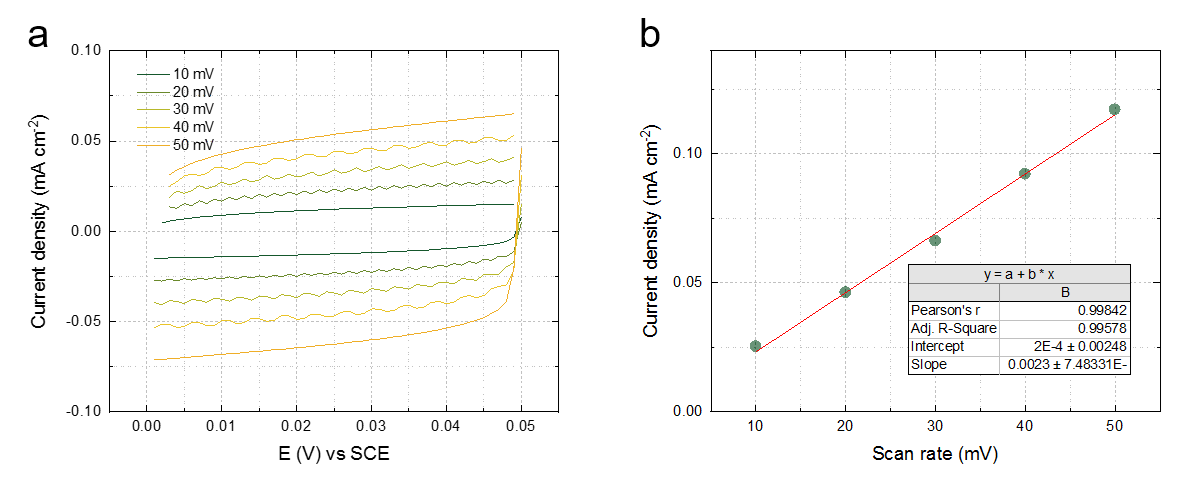


**Figure S49.** Double-layer capacitance measurements for determining electrochemically active surface area (ECSA) of La_2_NiFeO_6_ from voltammetry in 1.0 M KOH solution. (a) Typical cyclic voltammograms in the non-Faradaic region around 0.05 V relative to SCE at different scan rates (0.01, 0.02, 0.03, 0.04, 0.05 V s^-1^ ); (b) the average of the cathodic and anodic charging currents absolute values measured at 0.025 V (vs. SCE) plotted as a function of scan rate.

**Figure S50.** XRD patterns of Fe-LNO after different cycle times.

**Crystal information**

chemical_formula_iupac 'La16 Ni16 O72 Fe16 H'

chemical_formula_moiety 'La16 Ni16 O72 Fe16 H'

chemical_formula_sum 'La16 Ni16 O72 Fe16 H'

chemical_formula_weight 5208.038

space_group_IT_number 1

symmetry_cell_setting triclinic

symmetry_space_group_name_Hall 'P 1'

symmetry_space_group_name_H-M 'P 1'

cell_length_a 7.4131

cell_length_b 10.6202

cell_length_c 25.8982

cell_angle_alpha 90.0000

cell_angle_beta 94.6130

cell_angle_gamma 90.0000

cell_volume 2025.7389

Atom position:

| Atom | X | Y | Z |
| --- | --- | --- | --- |
| La | 0.194416 | 0.000000 | 0.031543 |
| La | 0.226534 | 0.250000 | 0.138572 |
| La | 0.257447 | 0.000000 | 0.244619 |
| La | 0.286229 | 0.238098 | 0.345447 |
| Ni | 0.437622 | 0.250000 | 0.007819 |
| Ni | 0.472057 | 0.000000 | 0.126430 |
| Ni | 0.004842 | 0.250000 | 0.238153 |
| Ni | 0.035100 | 0.999984 | 0.345738 |
| O | 0.453854 | 0.377887 | 0.066243 |
| O | 0.488449 | 0.126280 | 0.181193 |
| O | 0.043510 | 0.376458 | 0.293962 |
| O | 0.453854 | 0.1221130 | 0.066243 |
| O | 0.488449 | 0.373720 | 0.181193 |
| O | 0.025193 | 0.125304 | 0.292407 |
| O | 0.183966 | 0.250000 | 0.99469 |
| O | 0.22045 | 0.000000 | 0.120867 |
| O | 0.255306 | 0.250000 | 0.239723 |
| O | 0.284016 | 0.999973 | 0.338557 |
| Fe | 0.33222 | 0.120718 | 0.525655 |
| O | 0.047196 | 0.131814 | 0.398887 |
| O | 0.081720 | 0.122366 | 0.507543 |
| Fe | 0.065902 | 0.000067 | 0.452269 |
| O | 0.352193 | 0.999987 | 0.570576 |
| O | 0.321310 | 0.000047 | 0.465751 |
| Fe | 0.329643 | 0.373093 | 0.526088 |
| O | 0.02278 | 0.373486 | 0.402225 |
| O | 0.080496 | 0.375239 | 0.509642 |
| Fe | 0.069363 | 0.250789 | 0.455461 |
| O | 0.358388 | 0.247812 | 0.568061 |
| O | 0.322301 | 0.254257 | 0.465652 |
| La | 0.194416 | 0.500000 | 0.031543 |
| La | 0.226534 | 0.750000 | 0.138572 |
| La | 0.257447 | 0.500000 | 0.244619 |
| La | 0.286259 | 0.761867 | 0.345475 |
| Ni | 0.437622 | 0.750000 | 0.007819 |
| Ni | 0.472057 | 0.500000 | 0.12643 |
| Ni | 0.004842 | 0.750000 | 0.238153 |
| Ni | 0.033321 | 0.499978 | 0.347274 |
| O | 0.453854 | 0.877887 | 0.066243 |
| O | 0.488449 | 0.626279 | 0.181193 |
| O | 0.025236 | 0.874638 | 0.292422 |
| O | 0.453854 | 0.622113 | 0.066243 |
| O | 0.488449 | 0.873720 | 0.181193 |
| O | 0.043433 | 0.623470 | 0.293941 |
| O | 0.183966 | 0.750000 | 0.99469 |
| O | 0.22045 | 0.500000 | 0.120867 |
| O | 0.255306 | 0.750000 | 0.239723 |
| O | 0.290731 | 0.499934 | 0.360949 |
| Fe | 0.329681 | 0.626970 | 0.52615 |
| O | 0.022803 | 0.626621 | 0.402128 |
| O | 0.080531 | 0.624852 | 0.509706 |
| Fe | 0.058636 | 0.500136 | 0.45509 |
| O | 0.357631 | 0.499985 | 0.567758 |
| O | 0.321290 | 0.500082 | 0.465416 |
| Fe | 0.332200 | 0.879276 | 0.525625 |
| O | 0.047244 | 0.86824 | 0.398955 |
| O | 0.081706 | 0.877665 | 0.507502 |
| Fe | 0.069340 | 0.749107 | 0.455472 |
| O | 0.358367 | 0.752272 | 0.568101 |
| O | 0.322289 | 0.745724 | 0.465685 |
| La | 0.694416 | 0.000000 | 0.031543 |
| La | 0.726534 | 0.250000 | 0.138572 |
| La | 0.757447 | 0.000000 | 0.244619 |
| La | 0.787918 | 0.246931 | 0.350613 |
| Ni | 0.937622 | 0.250000 | 0.007819 |
| Ni | 0.972057 | 0.000000 | 0.126430 |
| Ni | 0.504842 | 0.250000 | 0.238153 |
| Ni | 0.537242 | 0.999973 | 0.345619 |
| O | 0.953854 | 0.377887 | 0.066243 |
| O | 0.988449 | 0.126280 | 0.181193 |
| O | 0.498656 | 0.376411 | 0.293938 |
| O | 0.953854 | 0.1221130 | 0.066243 |
| O | 0.988449 | 0.373720 | 0.181193 |
| O | 0.516118 | 0.1251900 | 0.292352 |
| O | 0.683966 | 0.250000 | 0.99469 |
| O | 0.720450 | 0.000000 | 0.120867 |
| O | 0.755306 | 0.250000 | 0.239723 |
| O | 0.783438 | 0.999905 | 0.335951 |
| Fe | 0.835823 | 0.1182610 | 0.526048 |
| O | 0.556133 | 0.1318120 | 0.398489 |
| O | 0.580533 | 0.122074 | 0.506691 |
| Fe | 0.567001 | 0.000092 | 0.452249 |
| O | 0.848195 | 0.999999 | 0.572876 |
| O | 0.820154 | 0.000037 | 0.467282 |
| Fe | 0.835167 | 0.379773 | 0.529073 |
| O | 0.583807 | 0.373831 | 0.402001 |
| O | 0.581998 | 0.376218 | 0.508668 |
| Fe | 0.568052 | 0.251034 | 0.455359 |
| O | 0.841712 | 0.246932 | 0.567803 |
| O | 0.822943 | 0.247817 | 0.470074 |
| La | 0.694416 | 0.500000 | 0.031543 |
| La | 0.726534 | 0.750000 | 0.138572 |
| La | 0.757447 | 0.500000 | 0.244619 |
| La | 0.787921 | 0.7531600 | 0.350606 |
| Ni | 0.937622 | 0.750000 | 0.007819 |
| Ni | 0.972057 | 0.500000 | 0.12643 |
| Ni | 0.504842 | 0.750000 | 0.238153 |
| Ni | 0.540196 | 0.499963 | 0.347194 |
| O | 0.953854 | 0.877887 | 0.066243 |
| O | 0.988449 | 0.626279 | 0.181193 |
| O | 0.516102 | 0.874713 | 0.292383 |
| O | 0.953854 | 0.622113 | 0.066243 |
| O | 0.988449 | 0.873720 | 0.181193 |
| O | 0.498721 | 0.623476 | 0.293908 |
| O | 0.683966 | 0.750000 | 0.99469 |
| O | 0.720450 | 0.500000 | 0.120867 |
| O | 0.755306 | 0.750000 | 0.239723 |
| O | 0.782340 | 0.499833 | 0.331663 |
| Fe | 0.835213 | 0.620282 | 0.529124 |
| O | 0.583705 | 0.626345 | 0.401845 |
| O | 0.582019 | 0.623891 | 0.508787 |
| Fe | 0.576818 | 0.500234 | 0.455167 |
| O | 0.850645 | 0.499985 | 0.575322 |
| O | 0.822416 | 0.500131 | 0.472304 |
| Fe | 0.835791 | 0.881761 | 0.526026 |
| O | 0.556138 | 0.868249 | 0.398601 |
| O | 0.580512 | 0.877924 | 0.506631 |
| Fe | 0.568078 | 0.74871 | 0.455381 |
| O | 0.841798 | 0.753149 | 0.567828 |
| O | 0.822952 | 0.752130 | 0.470080 |
| H | 0.301533 | 0.499967 | 0.399977 |

**References**

1. I. C. Man, H. Y. Su, F. Calle‐Vallejo, H. A. Hansen, J. I. Martínez, N. G. Inoglu, J. Kitchin, T. F. Jaramillo, J. K. Nørskov, J. Rossmeisl, *ChemCatChem* **2011**, *3*, 1159-1165.
2. L. C. Seitz, C. F. Dickens, K. Nishio, Y. Hikita, J. Montoya, A. Doyle, C. Kirk, A. Vojvodic, H. Y. Hwang, J. K. Norskov, *Science* **2016**, *353*, 1011-1014.
3. Kibsgaard, C. Tsai, K. Chan, J. D. Benck, J. K. Nørskov, F. Abild-Pedersen, T. F. Jaramillo, *Energy & Environmental Science* **2015**, *8*, 3022-3029.
